# Glutaminolysis drives membrane trafficking to promote invasiveness of breast cancer cells

**DOI:** 10.1038/s41467-017-02101-2

**Published:** 2017-12-21

**Authors:** Emmanuel Dornier, Nicolas Rabas, Louise Mitchell, David Novo, Sandeep Dhayade, Sergi Marco, Gillian Mackay, David Sumpton, Maria Pallares, Colin Nixon, Karen Blyth, Iain R. Macpherson, Elena Rainero, Jim C. Norman

**Affiliations:** 1 CRUK Beatson Institute for Cancer Research, Garscube Estate, Glasgow, G61 1BD UK; 2 Institute of Cancer Sciences, University of Glasgow, Glasgow, G61 1QH UK; 3 Biomedical Science Department, The University of Sheffield, Western Bank, Sheffield, S10 2TN UK

## Abstract

The role of glutaminolysis in providing metabolites to support tumour growth is well-established, but the involvement of glutamine metabolism in invasive processes is yet to be elucidated. Here we show that normal mammary epithelial cells consume glutamine, but do not secrete glutamate. Indeed, low levels of extracellular glutamate are necessary to maintain epithelial homoeostasis, and provision of glutamate drives disruption of epithelial morphology and promotes key characteristics of the invasive phenotype such as lumen-filling and basement membrane disruption. By contrast, primary cultures of invasive breast cancer cells convert glutamine to glutamate which is released from the cell through the system Xc- antiporter to activate a metabotropic glutamate receptor. This contributes to the intrinsic aggressiveness of these cells by upregulating Rab27-dependent recycling of the transmembrane matrix metalloprotease, MT1-MMP to promote invasive behaviour leading to basement membrane disruption. These data indicate that acquisition of the ability to release glutamate is a key watershed in disease aggressiveness.

## Abstract (web summary)

Glutamine metabolism is well known to support tumour growth. Here the authors show that cancer cells also utilize glutamine to promote invasiveness by converting it to glutamate, which upon secretion activates metabotropic glutamate receptors to stimulate matrix metalloproteases recycling to the cell surface.

## Introduction

Altered cell metabolism is a hallmark of cancer. Cancer cells have evolved a number of metabolic adaptations which enable them to grow and divide under conditions that are adverse to rapid cell proliferation^1^. Glucose and glutamine are key nutrients that provide energy and generate biosynthetic intermediates to produce macromolecules (amino acids and nucleotides) indispensable for proliferation. In addition to its function as a 'fuel', glutamine is also a key player in cytoprotective programmes that serve to 'buffer' insults encountered in the tumour microenvironment^2,3^. First, glutamine contributes to the synthesis of glutathione (a tri-peptide of glutamate, cysteine and glycine), an antioxidant molecule, by providing a source of glutamate that serves a substrate for glutamate-cysteine ligase. Secondly, glutamate allows import of cystine (another precursor of glutathione) via the system Xc- antiporter that is driven by equimolar export of glutamine-derived glutamate from the cell. Finally, glutamine-derived metabolites are substrates of malate dehydrogenase which generates NADPH, a molecule required to keep glutathione in its reduced form^2,3^.

In addition to uncontrolled cell growth and proliferation, carcinoma progression is accompanied by increased cell migration and invasion which drives cancer dissemination and metastasis^1^. An accepted watershed in breast cancer aggressiveness is the progression from ductal carcinoma in situ (DCIS), characterised by intraductal proliferation of malignant epithelial cells with an intact basement membrane, to invasive ductal carcinoma (IDC) in which the basement membrane becomes breached allowing dissemination of malignant cells^4^. Despite this, little is known about how altered energy metabolism of cancer cells might contribute to basement membrane disruption and subsequent migration of cancer cells from primary tumours. Clinical data indicate that expression of the ASCT2 transporter^5^ and system Xc- antiporter^6,7^ (controlling glutamine uptake and glutamate export respectively) are linked to metastasis and poor prognoses, indicating that metabolic adaptations adopted by cancer cells to support growth and to minimise oxidative stresses may also contribute to cancer aggressiveness. In this study we have found that high levels of glutamine consumption, in combination with functional expression of the system Xc- antiporter, contributes to cancer aggressiveness by generating a source of extracellular glutamate. This extracellular glutamate then activates the GRM3 metabotropic glutamate receptor to drive receptor recycling leading to basement membrane disruption and invasion in breast cancer.

## Results

### Glutamate release drives invasive behaviour

**Fig. 1:**
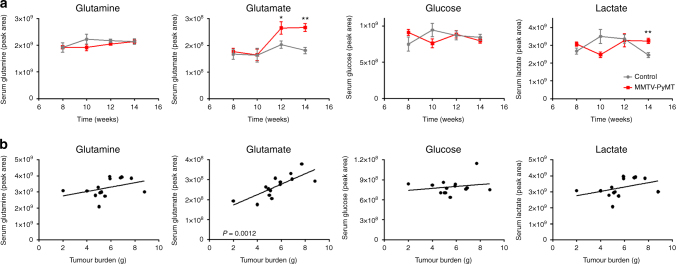
Serum glutamate levels reflect mammary tumour burden in MMTV-PyMT mice. FVB/N mice, carrying a mouse mammary tumour virus (MMTV) promoter-driven polyoma middle T (PyMT) transgene, were culled at 8, 10, 12 and 14 weeks of age and blood samples were collected via cardiac puncture. Serum was isolated and the levels of the indicated metabolites determined using mass spectrometry **a**. Primary breast tumour burden was assessed at the 14 week time point by determination of total mammary weight and these were plotted against the plasma levels of glutamine, glutamate, glucose and lactate **b**. Values are mean ± SEM, *p = 0.05, **p < 0.01, Mann–Whitney test

Expression of the polyoma middle T oncogene under control of the mammary epithelial MMTV promoter (MMTV-PyMT) provides a reliable model of breast cancer progression that recapitulates many aspects of the human disease^8^, in particular luminal B-type breast cancer^9^. To look for potential links between glutamine metabolism and breast tumour progression we measured levels of glutamine, glutamate and other metabolites in the serum of tumour-bearing MMTV-PyMT mice and compared these with non-tumour-bearing animals from the same genetic background. Furthermore, we investigated whether the levels of these circulating metabolites would correlate with mammary tumour burden. This indicated that serum glutamate levels (but not glutamine, glucose or lactate) become elevated in tumour-bearing animals over a time course that follows tumour progression (Fig. 1a), and that this correlates closely with tumour burden (Fig. 1b). In addition, we have measured the circulating levels of a broad range of metabolites during tumour progression in MMTV-PyMT mice, and found that glutamate is the only one whose serum levels positively correlate with primary mammary tumour burden.

**Fig. 2:**
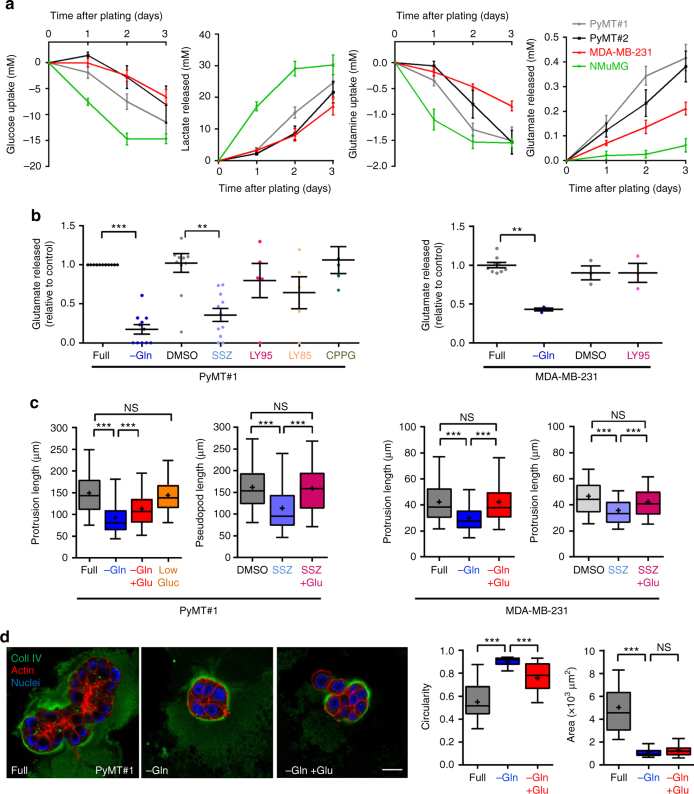
Glutamate release through the system Xc- antiporter drives invasive behaviour. **a**, **b** MMTV-PyMT tumour-derived cell lines from two different animals (PyMT#1, PyMT#2), MDA-MB-231 human breast cancer cells (MDA-MB-231) and normal murine mammary epithelial cells (NMuMG) were plated onto six-well dishes at 1 × 10^5^cells/well and grown towards confluence for 3 days. Aliquots of cell-exposed medium were collected at 24 h intervals and glucose, lactate, glutamine and glutamate concentration was determined. Values are mean ± SEM, *n* = 3 independent experiments. In **b** cells were incubated for 24 h in full DMEM (full), medium lacking glutamine (−Gln) or in the presence of sulphasalazine (SSZ; 200 μM), LY341495 (LY95; 100 nM), LY367385 (LY85; 15 μM), (RS)-α-Cyclopropyl-4-phosphonophenylglycine (CPPG; 5 nM) or DMSO control. Values are normalised to glutamate release in full DMEM. Mean and SEM are indicated, minimum of 3 independent experiments per conditions. ***p < 0.001, **p < 0.01 one-way ANOVA, Dunn’s multiple comparison test. **c** PyMT#1 or MDA-MB-231 cells were plated into fibroblast-derived extracellular matrix 4 h prior to imaging, in the presence of either full DMEM (full), glutamine-depleted medium (−Gln), glutamine-depleted medium supplemented with 10 µM glutamate (−Gln + Glu) or 1 mg/ml glucose-containing medium (Low gluc.) and imaged for 16 h by time-lapse microscopy. Where indicated, 200 μM sulphasalazine in the presence (SSZ + Glu) and absence (SSZ) of glutamate, or DMSO control were added to full DMEM. Protrusion length measured with Image J, *n* = 3 independent experiments (whiskers: 5–95 percentile, + represents the mean) (PyMT#1, full, −Gln, −Gln + Glu 540 cells; PyMT#1, DMSO, SSZ 360 cells, SSZ + Glu 200 cells, MDA-MB-231 full 338 cells, −Gln 349 cells, −Gln + Glu 354 cells; MDA-MB-231 DMSO, SSZ 358 cells, SSZ + Glu 123 cells) ***p < 0.001 one-way ANOVA Dunn’s multiple comparison test. **d** PyMT#1 cells were plated into Matrigel in full DMEM (full), glutamine-depleted medium (−Gln) or glutamine-depleted medium supplemented with 10 μM glutamate (−Gln + Glu) and allowed to form organoids for 4 days before fixation and visualisation of collagen IV (coll IV; green), F-actin (phalloidin; red) and cell nuclei (DAPI; blue) by confocal microscopy. Bar, 20 μm. The circularity and area of organoids was determined using Image J. Mean and SEM are indicated, *n* = 3 independent experiments (full 56 acini, −Gln 55 acini, −Gln + Glu 52 acini). ***p < 0.001, two-way ANOVA Dunn’s multiple comparison test

The tumour burden of MMTV-PyMT mice may constitute a substantial proportion of body weight suggesting the possibility that the source of elevated circulating glutamate may be the tumour itself. To investigate this, we generated two low-passage cell lines from MMTV-PyMT tumours (named PyMT#1 and PyMT#2), isolated from different animals, which are highly invasive in a range of *ex vivo* assays, form invasive tumours when transplanted into the mammary fat pads of recipient mice, and aggressively colonise the lung when introduced intravenously. We measured glucose/glutamine consumption and lactate/glutamate production in cells from invasive MMTV-PyMT tumours and compared these with normal mouse mammary epithelial cells (NMuMG), and the well-established triple-negative invasive human breast cancer cell line, MDA-MB-231. MMTV-PyMT tumour-derived cell lines, NMuMG and MDA-MB-231 cells all consumed glucose and glutamine (Fig. 2a). Moreover, both the normal and invasive cell types produced lactate at levels that were commensurate with their glucose consumption. Invasive cells (PyMT#1, PyMT#2 & MDA-MB-231) released glutamate at rates that also corresponded with their glutamine consumption, and this was opposed by glutamine withdrawal indicating that secreted glutamate is mostly derived from glutaminolysis (Fig. 2a, b). In striking contrast, glutamate did not accumulate in the medium surrounding breast epithelial cells (NMuMG) indicating that these normal cells had reduced capacity to release glutamate, despite their avid consumption of glutamine (Fig. 2a). MMTV-PyMT tumour-derived cell lines express the SLC7A11 subunit (also called xCT) of the system Xc- glutamate–cystine antiporter at significantly higher levels than NMuMG cells (Supplementary Fig. 1a), suggesting that acquisition of expression of this transporter might be responsible for increased glutamate release from invasive cells. Indeed, inhibition of the Xc- antiporter with sulphasalazine (SSZ) potently opposed glutamate release (Fig. 2b).

Breast cancer cells extend invasive protrusions when they migrate into a fibroblast-derived 3D extracellular matrix (ECM) microenvironment which resembles the tumour stroma^10^, and this key facet of invasiveness is opposed by pharmacological inhibition or siRNA of the transmembrane matrix metalloprotease, MT1-MMP—as described below. We used this quantitative and robust approach to investigate the contribution of glucose and glutamate metabolism to invasive behaviour. Whilst MMTV-PyMT tumour-derived cell lines extended invasive protrusions in medium with reduced glucose concentration, depletion of glutamine significantly reduced protrusion length indicating that glutaminolysis (but not glycolysis) contributes to invasiveness (Fig. 2c; Supplementary Fig. 1c). Moreover, addition of oligomycin did not affect protrusion length, indicating that the requirement for glutamine in invasiveness is not due to its role in the generation of Krebs cycle intermediates to fuel oxidative phosphorylation (Supplementary Fig. 1d). Furthermore, addition of SSZ to inhibit the system Xc- antiporter or siRNA of the Xc- subunit xCT strongly reduced protrusion length (Fig. 2c; Supplementary Fig. 1b). We, therefore, tested the requirement for extracellular glutamate in invasiveness and found that addition of glutamate significantly restored the ability of glutamine-deprived and SSZ-treated MMTV-PyMT and MDA-MB-231 cells to extend invasive protrusions (Fig. 2c). It is important to note that MDA-MB-231 and PyMT cells are unable to grow in the absence of glutamine (Supplementary Fig. 1e), and PyMT (but not MDA-MB-231) cells display moderately increased cell death following withdrawal of glutamine (Supplementary Fig. 1f). However, addition of glutamate at a concentration that modulates invasion did not influence cell death nor did it support growth of glutamine-deprived cells, indicating that any role played by extracellular glutamate under these conditions is mediated via control of the cell migration/invasion machinery and not by promoting cell growth or suppressing apoptosis (Supplementary Fig. 1e,f).

MMTV-PyMT tumour-derived cells formed irregularly-shaped organoids (with low circularity) in 3D cultures and although they assemble the vestiges of collagen IV-rich basement membranes, these structures are discontinuous and display marked breaches through which cancer cells leave the organoid and disseminate (Fig. 2d). When deprived of glutamine, however, PyMT cells formed smaller organoids (reflecting their requirement for glutamine to grow) bounded by an intact collagen-rich basement membrane with high circularity (Fig. 2d). Addition of glutamate to glutamine-starved organoids did not increase their size (consistent with the inability of glutamate to support growth in the absence of glutamine), but drove basement membrane disruption and migration of cells out of the organoid which is reflected by reduced circularity of the structure (Fig. 2d). Taken together, these data indicate that although glutamine is essential for efficient growth of tumour cells and to oppose apoptosis, their invasiveness is supported by an extracellular pool of glutamate generated by glutaminolysis followed by its system Xc- antiporter-mediated export into the extracellular milieu.

### Glutamate drives invasion through the GRM3 receptor

**Fig. 3:**
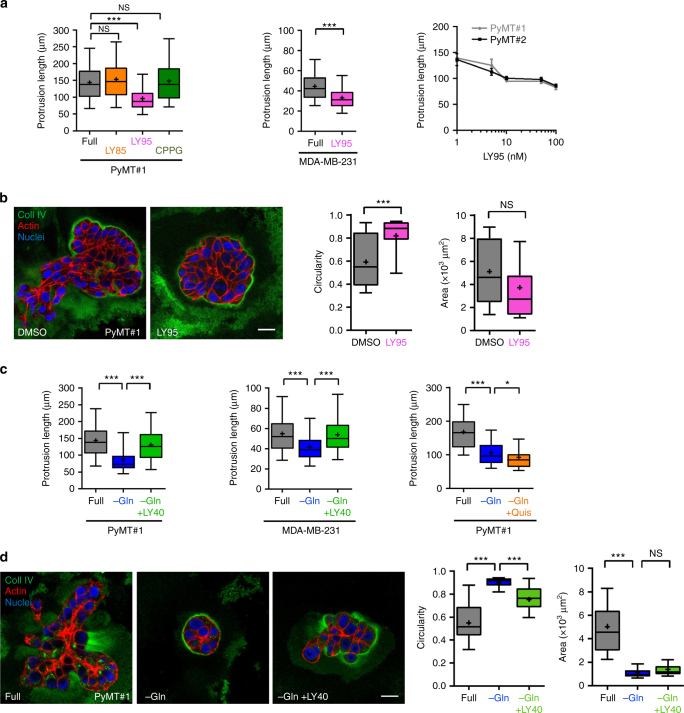
Extracellular glutamate drives invasion through a type II metabotropic receptor. **a** MMTV-PyMT#1 tumour-derived cell lines or MDA-MB-231 cells were plated into fibroblast-derived extracellular matrix in full DMEM (full), in the presence of LY341495 (LY95; 100 nM), LY367385 (LY85; 15 μM), or (RS)-α-Cyclopropyl-4-phosphonophenylglycine (CPPG; 5 nM) and invasive protrusion length determined as for Fig. 2c. Data are represented as box and whiskers plots (whiskers: 5–95 percentile, + represents the mean), *n* = 3 independent experiments (PyMT#1 360 cells per condition; MDA-MB-231 full 359 cells, LY95 358 cells) ***p < 0.001 one-way ANOVA, Dunn’s multiple comparison test (left graph) and Mann-Whitney test (centre graph). The right panel displays a dose-inhibition response curve to a range of LY341495 (LY95) concentrations plotted on a log_10_ axis, *n* = 3 independent experiments (360 cells per condition), data are mean ± SEM. **b** PyMT#1 cells were plated into 3D Matrigel cultures in the presence of LY341495 (LY95; 100 nM) or DMSO control. After 4 days, cells were fixed and collagen IV (coll IV; green), F-actin (phalloidin; red) and cell nuclei (DAPI; blue) visualised by immunofluorescence followed by confocal microscopy. The circularity and area of the organoids was determined as for Fig. 2d. Bar, 20 μm. The mean and SEM are indicated, *n* = 3 independent experiments (DMSO 37 acini, LY95 42 acini). ***p < 0.001, Mann–Whitney test. **c** The length of invasive protrusions emanating from PyMT#1 and MDA-MB-231 cells was determined as for (**a**) in full DMEM (full), or in glutamine-depleted medium (−Gln) in the absence and presence of LY354740 (LY40; 50 nM) or quisqualate (Quis; 1 μM). Data are represented as box and whiskers plots (whiskers: 5–95 percentile, + represents the mean), *n* = 3 independent experiments (PyMT#1 360 cells; MDA-MB-231 350 cells per condition) ***p < 0.0001 one-way ANOVA, Dunn’s multiple comparison test. **d** PyMT#1 cells were plated into Matrigel in full DMEM (full), glutamine-depleted medium (−Gln) or glutamine-depleted medium supplemented with LY354740 (LY40; 50 nM) and allowed to form organoids for 4 days. Immunofluorescence and quantification is as for (b). Bar, 20 μm. The mean and SEM are indicated, *n* = 3 independent experiments (full 46 acini, −Gln 45 acini, −Gln + LY40 43 acini). ***p < 0.001, two-way ANOVA, Dunn’s multiple comparison test

Metabotropic glutamate receptors (GRMs), are a family of G-protein-coupled receptors established to mediate responses to extracellular glutamate in the brain^11^, and these receptors have also been linked to growth of malignant brain tumours (such as glioma^12,13^) and other cancer types (for instance breast^14^ and melanoma^15,16^). There are three groups of GRMs—groups I, II and III. MMTV-PyMT tumour-derived cell lines, MDA-MB-231 cells and normal mammary epithelial cells express representatives of each of these receptor subclasses (Supplementary Fig. 2a). We tested the ability of inhibitors selective for these receptor subclasses to oppose extension of invasive protrusions in glutamine-containing medium. An inhibitor of group II GRM receptors (LY341495; subsequently referred to as LY95) strongly inhibited extension of invasive protrusions in the low nM range (consistent with the K_i_ of this compound for group II GRMs), whilst antagonists of group I & III (LY367385 (LY85) & CPPG respectively) receptor subclasses were ineffective in this regard (Fig. 3a and Supplementary Fig. 2b). Consistently, following blockade of group II GRMs (with LY95), PyMT cells grew in 3D cultures as large spherical organoids (with high circularity) surrounded by an intact collagen IV-rich basement membrane (Fig. 3b). Furthermore, as the PyMT model most closely matches the luminal B type of breast cancer, we used the ZR75-1 human cell line which is derived from this form of the disease. ZR75-1 cells secrete glutamate^17^ (Supplementary Fig. 3a), express GRM3 (Supplementary Fig. 3b) and grew as irregularly-shaped organoids in 3D cultures that displayed extensively disrupted basement membranes (Supplementary Fig. 3d). Addition of LY95 to these cultures significantly opposed this invasive morphology and led to generation of spherical organoids surrounded by a basement membrane, indicating that group II GRMs contribute to invasiveness in this luminal B-type human cell line (Supplementary Fig. 3d).

Since blockade of group II GRMs opposes the invasive behaviour of breast cancer cells, we then investigated the ability of a group II GRM agonist to promote invasiveness. Indeed, activation of group II GRMs with LY40 (but not activation of group I GRMs with quisqualic acid), was sufficient to restore extension of invasive protrusions (Fig. 3c and Supplementary Fig. 2c) and to drive basement membrane disruption and cell dissemination without rescuing the impaired growth or influencing apoptosis in glutamine-starved conditions (Fig. 3d; Supplementary Fig. 1e, f).

**Fig. 4:**
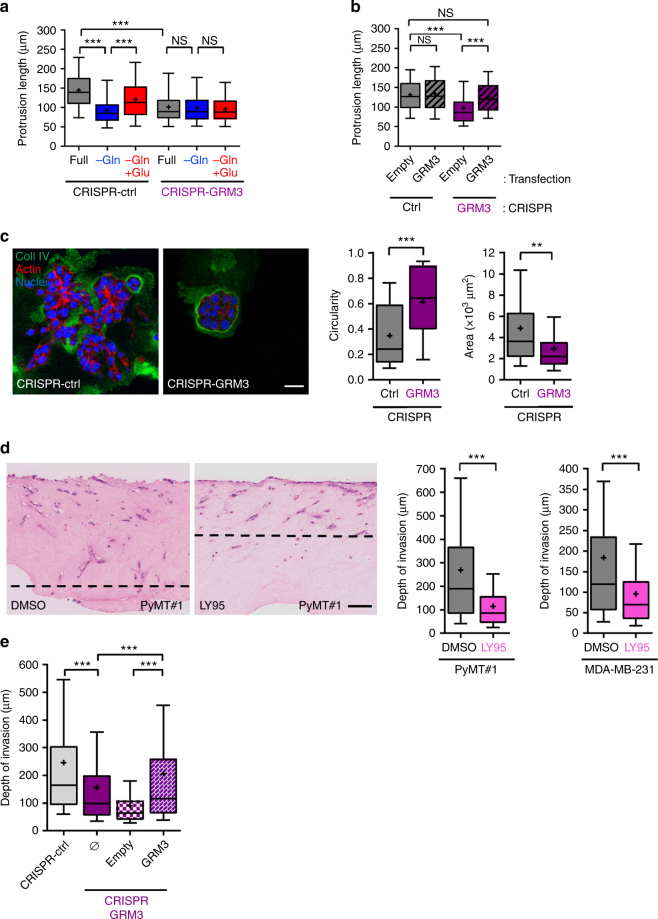
The GRM3 metabotropic receptor is required for glutamate-driven invasiveness. **a** An MMTV-PyMT tumour-derived cell line in which GRM3 expression had been disrupted by CRISPR gene editing (CRISPR-GRM3) and a corresponding pool that had been transfected with non-targeting CRISPR guides (CRISPR-ctrl) were generated. CRISPR-GRM3 and CRISPR-ctrl cells were plated into fibroblast-derived ECM to determine their ability to extend invasive protrusions and these were analysed as for Fig. 2c. Data are represented as box and whiskers plots (whiskers: 5–95 percentile, + represents the mean), *n* = 3 independent experiments (300 cells per condition). ***p < 0.001 one-way ANOVA, Dunn’s multiple comparison test. **b** CRISPR-Ctrl and CRISPR-GRM3 cells containing an expression vector encoding flag-tagged GRM3 (GRM3) or empty vector control (empty) were plated onto fibroblast-derived ECM and their ability to extend invasive protrusions was determined as for Fig. 2c. Data are represented as box and whiskers plots (whiskers: 5–95 percentile, + represents the mean), *n* = 3 independent experiments (300 cells per condition). ***p < 0.001 one-way ANOVA, Dunn’s multiple comparison test. **c** CRISPR-Ctrl and CISPR-GRM3 cells were grown in 3D Matrigel cultures and their morphology and basement membrane integrity determined as for Fig. 2d. Bar, 20 μm. The mean and SEM are indicated, *n* = 3 independent experiments. ***p < 0.001, **p < 0.01, one-way ANOVA, Dunn’s multiple comparison test. **d** PyMT#1 or MDA-MB-231 cells were plated onto a collagen plug that had been pre-conditioned by primary human fibroblasts and allowed to invade for 6 days in the presence and absence of LY341495 (LY95; 100 nM) or DMSO control. Scale bar, 100 μm. At least 6 individual plugs per condition were analysed in 3 independent experiments and the depth of cells invading was determined for each plug. Data are represented as box and whiskers plots (whiskers: 5–95 percentile, + represents the mean), *n* = 3 independent experiments ***p < 0.001 two-sided Mann–Whitney test. **e** CRISPR-Ctrl and CRISPR-GRM3 cells containing an expression vector encoding flag-tagged GRM3 (GRM3) or empty vector control (empty) were plated onto fibroblast pre-conditioned collagen plugs and their invasion into these determined as for (d). Data are represented as box and whiskers plots (whiskers: 5–95 percentile, + represents the mean), *n* = 3 independent experiments *** *p* < 0.001 2-sided Mann-Whitney test

GRM3 is the only member of the group II receptor subclass expressed by MMTV-PyMT tumour-derived cell lines and MDA-MB-231 cells (Supplementary Fig. 2a). We confirmed that MDA-MB-231 cells expressed GRM3 using western blotting (Supplementary Fig. 2d). However, we have been unable to obtain an antibody capable of detecting the mouse GRM3 protein. We tested the involvement of GRM3 in invasive responses by generating MMTV-PyMT tumour-derived cells in which GRM3 expression is disrupted by CRISPR gene editing (CRISPR-GRM3) (Supplementary Fig. 4a, b). CRISPR-GRM3 cells were completely unable to extend invasive protrusions in response to addition of glutamate (Fig. 4a, b), and grew as non-invasive organoids with high circularity surrounded by collagen IV-rich basement membranes when plated into 3D cultures (Fig. 4c). By contrast, an MMTV-PyMT tumour-derived cell line that had been subject in parallel to non-targeting CRISPR guides (CRISPR-ctrl) was highly invasive in both these assays. Importantly, expression of flag-tagged human GRM3 (Supplementary Fig. 4c) completely restored the ability of CRISPR-GRM3 cells to extend invasive protrusions whilst having no influence on the protrusion length of CRISPR-ctrl cells (Fig. 4b).

To further investigate the role of GRMs in breast cancer invasive behaviour, we established organotypic cultures in which tumour cells were plated onto the air-liquid interface of a collagen type I matrix that had been preconditioned by human epidermal fibroblasts, an approach that recapitulates many of the key aspects of the stromal microenvironment^18,19^. Inhibition of GRM3 with LY95 significantly opposed the ability of MMTV-PyMT tumour-derived cell lines and MDA-MB-231 cells to invade this 3D microenvironment (Fig. 4d). Furthermore, disruption of GRM3 using CRISPR significantly opposed the ability of MMTV-PyMT tumour-derived cells to invade into organotypic plugs, and this was completely rescued by expression of flag-tagged human GRM3 (Fig. 4e).

### GRM3 drives invasion via Rab27-dependent MT1-MMP recycling

**Fig. 5:**
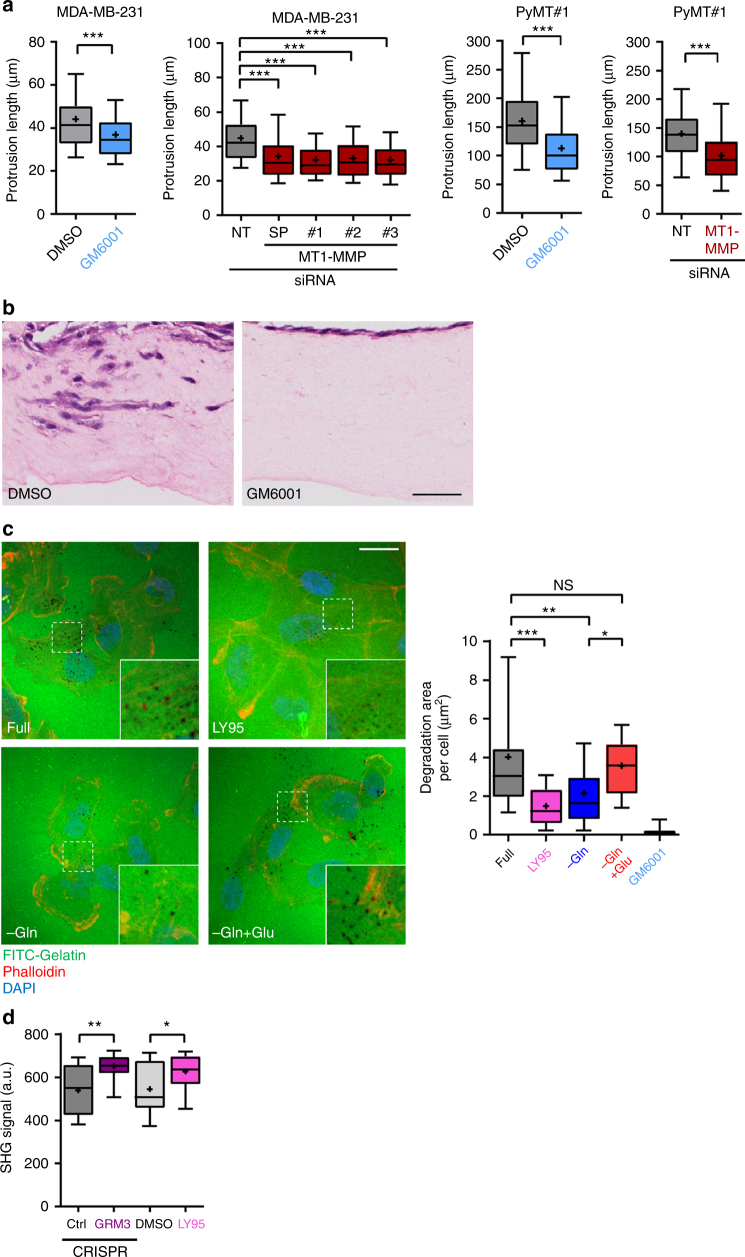
GRM3 drives MT1-MMP-dependent ECM degradation. **a** MDA-MB-231 cells or MMTV-PyMT#1 tumour-derived cells were transfected with siRNAs targeting MT1-MMP (SMARTPool (SP) or individual oligos) or non-targeting control (NT). 24 h after transfection, cells were plated into fibroblast-derived extracellular matrix in full DMEM in the presence of GM6001 (5 μM) and invasive protrusion length determined as for Fig. 2c. Data are represented as box and whiskers plots (whiskers: 5–95 percentile, + represents the mean), *n* = 3 independent experiments (PyMT#1 240 cells per condition; MDA-MB-231 245 cells per condition) ***p < 0.001 one-way ANOVA, Dunn’s multiple comparison test. **b** MMTV-PyMT#1 cells were plated onto a collagen plug that had been pre-conditioned by primary human fibroblasts and allowed to invade for 6 days in the presence and absence of GM6001 (5 μM) or DMSO control. Image representative of *n* = 2 independent experiments. Bar, 100 μm. **c** MDA-MB-231 cells were plated onto glass surfaces that had been pre-coated with FITC-conjugated gelatin (green signal) in either full DMEM (full)—in the presence and in the absence of LY341495 (LY95; 100 nM) or GM6001 (5 μM)—or in glutamine-depleted medium (−Gln), or glutamine-depleted medium supplemented with 10 µM glutamate (−Gln + Glu). 16 h following plating, cells were fixed and stained with phalloidin (red signal) and DAPI (blue signal) and visualised using fluorescence confocal microscopy. Bar, 20 μm. The area of degraded gelatin (black signal) was determined using Image J and corrected for cell number. Data are represented as box and whiskers plots (whiskers: 5–95 percentile, + represents the mean), *n* = 3 independent experiments ( > 40 cells per condition) ***p < 0.001, **p < 0.01, *p < 0.05 one-way ANOVA, Dunn’s multiple comparison test. **d** PyMT#1, CRISPR-Ctrl., and CRISPR-GRM3 cells were plated onto a collagen plug that had been pre-conditioned by primary human fibroblasts and allowed to invade for 6 days in the presence and absence of LY341495 (LY95; 100 nM) or DMSO control. The plugs were then imaged using second harmonic generation (SHG) microscopy to visualise fibrillar collagen. The level of the SHG signal was then calculated using image J. Data are represented as box and whiskers plots (whiskers: 5–95 percentile, + represents the mean), *n* = 3 independent experiments (18 plugs per condition) **p < 0.01, *p < 0.05 one-way ANOVA, Dunn’s multiple comparison test. Bar, 37.5 μm

The transmembrane matrix metalloprotease, MT1-MMP is a key initiator of proteolytic cascades that lead to invasiveness in cancer. Indeed, MT1-MMP becomes concentrated in protrusive ‘invadopodial’ structures which are involved with the initial penetration, destabilisation and breaching of collagen-rich basement membranes surrounding epithelia^20^. Consistently, suppression of MT1-MMP using inhibitors (GM6001) or siRNA strongly suppressed extension of invasive protrusions when MMTV-PyMT or MDA-MB-231 cells were plated into fibroblast-derived ECM (Fig. 5a; Supplementary Figure 5a, b), and completely opposed invasion of MMTV-PyMT cells into organotypic plugs (Fig. 5b). To directly address the possibility that GRM3 promotes ECM degradation and/or remodelling, we plated MDA-MB-231 cells onto a thin layer of fluorescently-labelled gelatin and measured the appearance of degraded foci (which correspond to active invadopodia) underneath the cells. Consistent with previous reports, addition of the MMP inhibitor GM6001 completely opposed degradation of gelatin by MDA-MB-231 cells (Fig. 5c). Importantly, withdrawal of glutamine or addition of LY95 also strongly opposed gelatin degradation, and addition of glutamate completely restored the ability of glutamine-starved cells to digest the fluorescent gelatin matrix (Fig. 5c). Finally, we used second harmonic generation (SHG) microscopy to determine the amount of fibrillar collagen in organotypic plugs containing MMTV-PyMT cells and found that they degrade the ECM to generate areas with reduced fibrillar collagen content (Fig. 5d). CRISPR-mediated disruption or pharmacological blockade of GRM3 using LY95 significantly opposed the ability of MMTV-PyMT cells to reduce the amount of fibrillar collagen in organotypic plugs indicating that GRM3 is required for breast cancer cells to efficiently degrade the ECM in a 3D microenvironment (Fig. 5d).

**Fig. 6:**
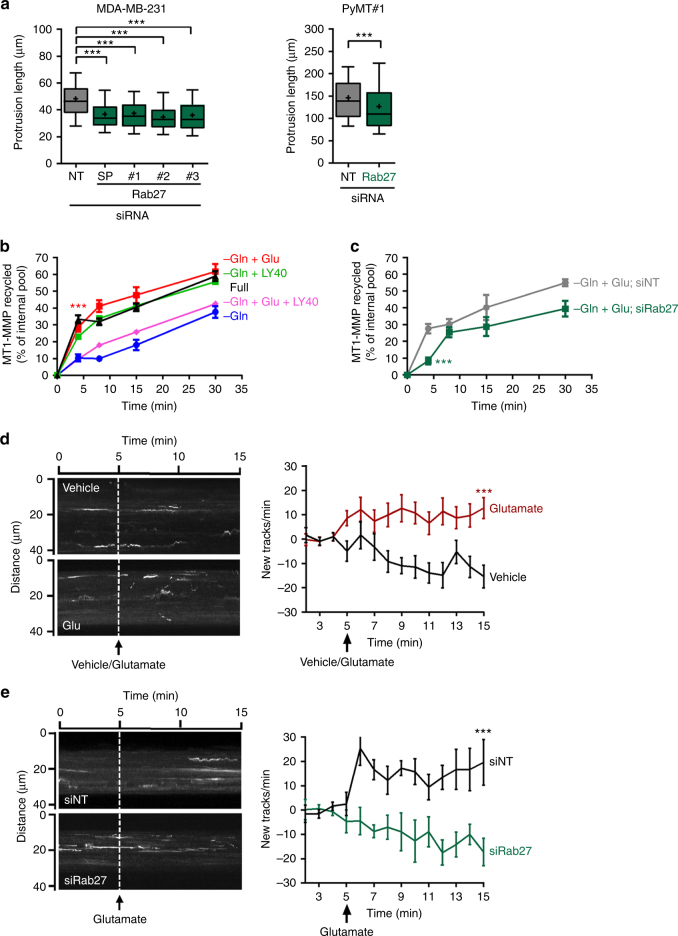
Extracellular glutamate drives Rab27-dependent recycling of MT1-MMP. **a** MDA-MB-231 cells and MMTV-PyMT tumour-derived cells (PyMT#1) transfected with siRNAs targeting Rab27 (SMARTPool (SP) or individual oligos) or non-targeting control (NT) were plated 24 h after transfection into fibroblast-derived extracellular matrix in full DMEM and invasive protrusion length determined as for Fig. 2c. Data are represented as box and whiskers plots (whiskers: 5–95 percentile, + represents the mean), *n* = 3 independent experiments (PyMT#1 > 200 cells; MDA-MB-231, > 250 cells) *** *p* < 0.001 **p < 0.01 one-way ANOVA, Dunn’s multiple comparison test. **b**, **c** MDA-MB-231 cells were transfected with siRNAs targeting Rab27 (siRab27) or non-targeting control (siNT) (**c**), or left untransfected (**b**). 96 h following transfection, cells were starved of glutamine for 90 min then surface-labelled with 0.13 mg/ml NHS-S-S-Biotin at 4 °C and internalisation was then allowed to proceed for 30 min at 37 °C. Biotin remaining at the cell surface was removed by exposure to MesNa at 4 °C, and internalised MT1-MMP chased back to the cell surface at 37 °C for the indicated times in the absence (−Gln) or presence of 15 μM glutamate (−Gln + Glu), or 50 nM LY354740 (−Gln + LY40) or glutamine-deprived media supplemented with 15 μM glutamate in the presence of 100 nM LY341495 (−Gln + Glu + LY95) or in full DMEM (full). Cells were then re-exposed to MesNa and biotinylated MT1-MMP determined by capture-ELISA with anti-human MT1-MMP antibodies. The proportion of MT1-MMP recycled to the plasma membrane is expressed as % of the pool of MT1-MMP labelled during the internalisation period. *n* = 3 independent experiments for full, –Gln and –Gln + Glu, values are mean ± SEM; *n* = 2 for –Gln + LY40 and –Glu + Glu + LY95, values are mean; *n* = 3 for –Glu + Glu; siNT and –Glu + Glu; siRab27, values are mean ± SEM. ***p < 0.001 Dunn’s multiple comparison test. **d**,**e** MDA-MB-231 cells transfected with mCherry-tagged-MT1-MMP in combination with siRNAs targeting Rab27 (siRab27) or non-targeting control (siNT) as indicated, and plated onto fibronectin-coated glass-bottom dishes. 18 h following transfection, medium changed for DMEM lacking serum and glutamine and cells imaged by TIRF microscopy. Frames were collected every 1.5 s, and glutamate (20 μM) or vehicle added at the indicated time. Image J (TrackMate plugin) was used for quantification. Values were normalised by subtracting the average rate 1–2 min prior to vehicle or glutamate addition. Kymographs display time on the horizontal axis and distance on the vertical axis. Values are mean ± SEM; *n* = 3 independent experiments. *** *p* < 0.001 two-way ANOVA, Dunn’s multiple comparison test

Endo-exocytic receptor trafficking plays a key role in cancer progression^21^. Regulation of endocytosis and recycling of integrin and receptor tyrosine kinases contributes to the ability of oncogenes, such as mutated forms of p53, to drive invasion and metastasis^22,23^. Membrane trafficking events are involved in the breaching of basement membranes during normal tissue morphogenesis, and DCIS to invasive tumour transitions in cancer^24,25^. MT1-MMP function is influenced by how it is trafficked through the endosomal system^26^. A number of factors control MT1-MMP trafficking, and recently the Rab27 GTPase was shown to be essential for the recycling of internalised MT1-MMP from late endosomes to the plasma membrane to promote invasiveness^24^. Knockdown of Rab27 opposed the ability of both MMTV-PyMT and MDA-MB-231 cells to extend invasive protrusions (Fig. 6a; Supplementary Fig. 5c, d), suggesting that glutamate-driven receptor trafficking might influence invasiveness through this GTPase. We, therefore, determined the influence of glutamine metabolism on recycling of MT1-MMP, and whether this is mediated via engagement of GRM3. In glutamine-starved cells, internalised MT1-MMP was recycled slowly to the plasma membrane (Fig. 6b). However, addition of glutamate or the GRM3 agonist, LY40, drove a ~5 fold increase in the rate of MT1-MMP recycling (Fig. 6b). Importantly, this was opposed following blockade of GRM3 with LY95 and the rate of MT1-MMP recycling became comparable to that of glutamine-starved conditions (Fig. 6b; Supplementary Fig. 3c). Furthermore, siRNA of Rab27 significantly slowed glutamate-driven recycling of MT1-MMP, and this was particularly apparent within 4 min of glutamate addition (Fig. 6c). To investigate the kinetics of MT1-MMP recycling in greater detail, we expressed mCherry-tagged MT1-MMP in MDA-MB-231 cells and used total internal reflectance fluorescence (TIRF) microscopy followed by image analysis to quantify the arrival of MT1-MMP-containing vesicles at the ventral plasma membrane. Addition of glutamate, compared to vehicle treated controls, drove a significant increase in the recruitment of MT1-MMP-positive vesicles to the ventral plasma membrane ~2–4 min following treatment (Fig. 6d). This was completely opposed by siRNA-mediated knockdown of Rab27 (Fig. 6e).

### Glutamate drives invasiveness of mammary epithelial cells

**Fig. 7:**
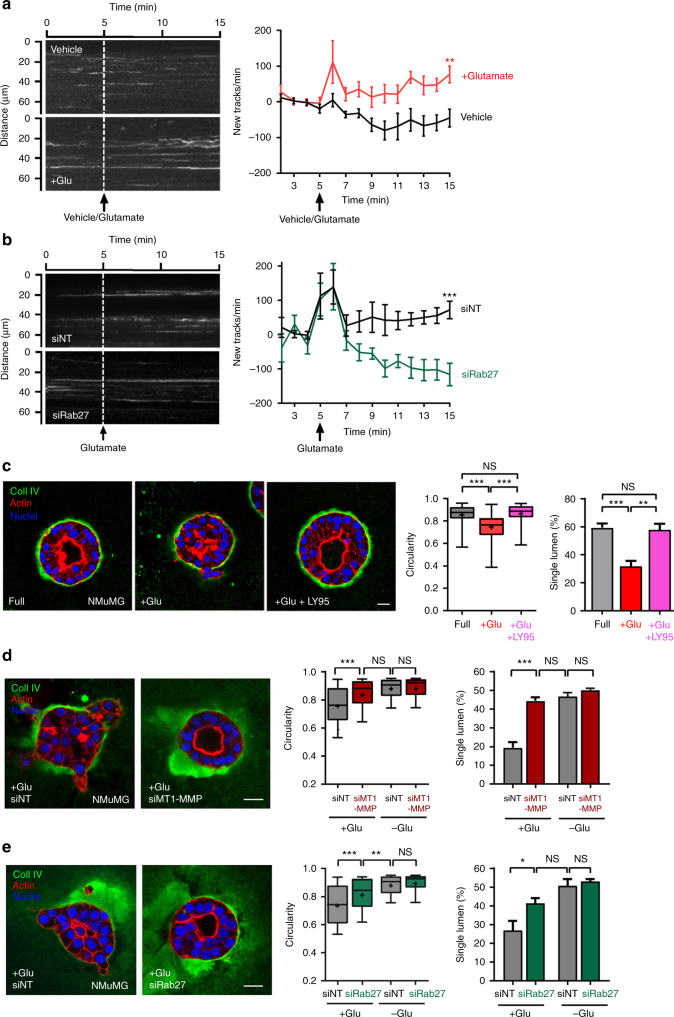
Normal mammary epithelial cells respond to extracellular glutamate by mobilising MT1-MMP to drive an invasive morphology. **a**, **b** Normal mammary epithelial (NMuMG) cells were transfected with mCherry-tagged MT1-MMP in combination with siRNAs targeting Rab27 (siRab27) or non-targeting control (siNT) as indicated, and plated onto Matrigel-coated glass-bottomed dishes. The rate of recruitment of vesicle positive for mCherry-MT1-MMP to the TIRF field was determined as for Fig. 6d, e. ***p < 0.001 **p < 0.01 two-way ANOVA, Dunn’s multiple comparison test. **c**,**e** NMuMG cells were transfected with siRNAs targeting MT1-MMP (siMT1-MMP), Rab27 (siRab27) or non-targeting control (siNT) as indicated (**d**, **e**) or were left untransfected (c). Cells were plated into 3D Matrigel plugs containing 1.6 mg/ml rat tail collagen I. Following culture in full DMEM for 4 days, the medium was changed for full DMEM or full DMEM supplemented with 10 μM glutamate with or without LY341495 (LY95; 100 nM) and cultured for a further 4 days. Immunofluorescence and circularity measurements were as for Fig. 2d. Bar, 20 μm. Circularity data are represented as box and whiskers plots (whiskers: 5–95 percentile, + represents the mean), *n* = 3 independent experiments ***p < 0.001 **p < 0.01 *p < 0.05 one-way ANOVA, Dunn’s multiple comparison test (full 97 acini, + Glu 80 acini, + Glu + LY95 94 acini; siNT + Glu 101 acini, siMT1-MMP + Glu 109 acini, siMT1-MMP-Glu 72, acini, siRab27 + Glu 68 acini, siRab27-Glu 69 acini). The proportion of organoids displaying single lumen was determined by examination of the F-actin distribution across a series of *Z*-planes using confocal microscopy. Values are mean ± SEM, *n* = 3 independent experiments. ***p < 0.001 **p < 0.01 one-way ANOVA Tukey’s comparison test

Normal mammary epithelial cells express low levels of xCT and secrete far less glutamate compared to invasive MMTV-PyMT and MDA-MB-231 cells (Fig. 2a; Supplementary Fig. 1a). However, because normal mammary epithelial cells express GRM3 (Supplementary Fig. 2a), it is possible that they are able to respond to extracellular glutamate by recycling MT1-MMP and mounting an invasive response. We transfected NMuMG cells with mCherry-tagged MT1-MMP and monitored its delivery to the plasma membrane using TIRF microscopy. Addition of glutamate to NMuMG cells led to a significant increase in recruitment of MT1-MMP vesicles to the plasma membrane (Fig. 7a) which was opposed by siRNA of Rab27 (Fig. 7b). In 3D cultures, mammary epithelial cells polarise to form spherical organoids (with high circularity) encircling a well-defined internal lumen (~60% of organoids possess a single lumen) bounded externally by a collagen IV-rich basement membrane (Fig. 7c). However, addition of glutamate led to discontinuities and breaches in the basement membrane with cells invading into the surrounding ECM (Fig. 7c). More surprisingly perhaps, addition of extracellular glutamate perturbed mammary epithelial polarisation as evidenced by a loss of single lumen structures (Fig. 7c). The ability of glutamate to drive basement membrane disruption and lumen-filling in NMuMG cells was completely dependent on GRM3 activity since this was reversed following blockade with LY95. Furthermore, MT1-MMP inhibition (with GM6001) or siRNA of MT1-MMP or Rab27 were sufficient to protect NMuMG cells from the transforming effects of glutamate (Fig. 7d,e; Supplementary Fig. 6a–c). Taken together, these data indicate that extracellular glutamate acts via GRM3 to promote invasive phenotypes in normal mammary epithelial cells by upregulating Rab27-dependent recycling of MT1-MMP.

## Discussion

**Fig. 8:**
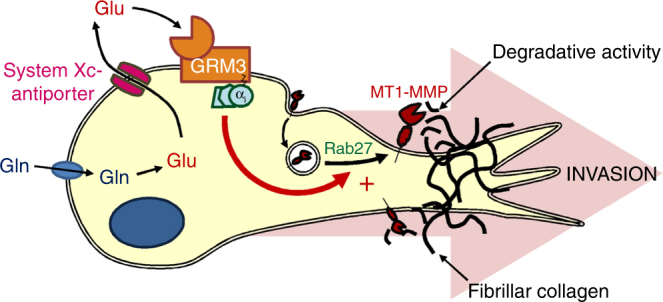
Glutaminolysis promotes invasiveness by providing a source of extracellular glutamate to activate GRM3-driven trafficking of MT1-MMP. In invasive cancer cells, glutamate (Glu) is generated from glutamine (Gln) by glutaminolysis and then released via the System Xc- antiporter to accumulate in the extracellular milieu. Extracellular glutamate can then activate the GRM3 metabotropic glutamate receptor. This fosters Rab27-dependent delivery of MT1-MMP to the plasma membrane where it mediates degradation of fibrillar collagen present within the extracellular matrix. Taken together, these processes lead to increased invasiveness that allows cancer cells to breach basement membrane and penetrate the ECM

Here we have shown that glutaminolysis can drive cancer invasiveness by increasing glutamate levels in the extracellular milieu. This, in turn, activates a metabotropic glutamate receptor to promote trafficking of a pro-invasive transmembrane matrix metalloprotease (Fig. 8). Cells from the normal mammary epithelium and those from invasive breast tumours consume glutamine at similar rates indicating that it is not upregulation of glutaminolysis itself that promotes invasiveness. Rather, acquisition of the expression of Xc- antiporter subunit xCT and release of glutamate through this channel dictates the transition between indolent and aggressive behaviours. The system Xc- antiporter is a key target of a cytoprotective/antioxidant transcriptional programme controlled by the NRF2 transcription factor^27^, and its role in this regard is to uptake cystine to support increased glutathione synthesis. Such cytoprotective programmes are commonly upregulated in cancer and this is thought to engender fast growing and chemoresistant tumours^28^. Because system Xc- antiporter also exports glutamate, an additional consequence of the NRF2 programme would be increased GRM3-driven invasiveness. Targeting cytoprotective programmes is being considered as a strategy for opposing tumour growth, but our findings indicate that blockade of system Xc- antiporter may also be effective in reducing tumour aggressiveness and this should be considered when drugs such as SSZ are evaluated clinically.

GRM signalling has been linked to pathogenesis in a number of cancer types, including brain tumours^12,13,29,30^, melanoma^16^ and breast cancer^14,15,31^. These studies have mostly linked activation of signalling downstream of GRMs (for example, protein kinase C, PI-3 kinase and MAP kinases) to cell growth and inhibition of apoptosis. Additionally, mutations in GRM3 that lead to constitutive receptor activation have been shown to provide cell proliferation and survival signals in melanoma^16^. Throughout these reports there are indications that cell migration may be affected by activation of GRM signalling, but the mechanisms that mediate this and whether this is linked to invasive behaviour is not clear. Although we have not been able to demonstrate any clear connection between extracellular glutamate and breast tumour cell growth or resistance to apoptosis, we find that the GRM3 metabotropic glutamate receptor is intimately linked to invasive behaviour. Moreover, we show for the first time that GRM signalling can influence vesicular trafficking and, specifically, that GRM3 activation fosters the endosomal trafficking of a pro-invasive transmembrane matrix metalloprotease—a process dependent on Rab27, a GTPase linked to poor prognosis and metastasis in breast cancer and glioma^32,33^. In the brain, ligation of GRM3 influences presynaptic function by suppressing the activity of adenylate cyclase to decrease cAMP formation and PKA activation^34^. This raises the fascinating possibility that cAMP signalling is a negative regulator of invasiveness in breast cancer, and further work will be necessary to elucidate how this is linked to trafficking of MT1-MMP and possibly to other pro-invasive receptors that cycle through the endosomal system.

Circulating glutamate levels have been known for some time to be elevated in breast and prostate cancer patients and have been linked to poor prognosis^35,36^. Indeed, we have found that glutamate (but not glutamine, glucose or lactate) levels increase significantly in the serum of tumour-bearing MMTV-PyMT mice by comparison with non-tumour-bearing control animals, and this correlates temporally with tumour progression and tumour burden. These data indicate a causal relationship between breast tumour burden and circulating glutamate concentration. However, the provenance of increased serum glutamate in cancer patients is not clear. The relationship between serum and intratumoural metabolite levels is unlikely to be straightforward, and currently it is technically challenging to measure the extracellular concentration of glutamate within tumours and tissues. In the brain, glutamate released by glutamatergic neurones is removed from the synaptic cleft and extrasynaptic sites via uptake into glial cells^37^, and it is possible that mechanisms exist in epithelia to sequester glutamate to avoid activation of GRMs on epithelial cell that could lead to dysplasia. Furthermore, stromal cells may influence glutamate levels in tumours and we have already observed that carcinoma-associated fibroblasts are capable of releasing considerable quantities of glutamate, and this would be likely to contribute to the stromal drive to tumour invasiveness. Finally, because GRM3 is expressed in a number of non-epithelial cell types it is probable that increased serum glutamate levels occurring in tumour-bearing animals may influence metastasis by activating GRM3 in immune cells, such as neutrophils and macrophages, that contribute to the seeding of metastases. We had approached this question by injecting MMTV-PyMT cells into the tail vein of mice and monitoring the ability of these to colonise the lung. We found that, although treatment of mice with LY95 significantly opposed lung colonisation (Supplementary Fig. 7), deletion of GRM3 in the MMTV-PyMT cells by CRISPR was not reproducibly effective in this regard. This indicates that, although GRM3 directly contributes to invasiveness of breast tumour cells and their ability to breach basement membranes, the contribution made by GRM3 to extravasation and metastatic seeding of disseminated cancer cells is more likely to be mediated via effects on other cell types.

Overall, our study has identified a new link between tumour cell metabolism and the invasive behaviour of tumour cells, and indicates that drugs capable of interfering with glutamate release or GRM receptor function may be useful in combatting tumour progression. We also shed light on a new link between GRM signalling and vesicular trafficking, although more work is required to understand how GRM downstream signalling impacts the endosomal recycling machinery. Future work will be required to accurately determine the extracellular concentrations of glutamate within tumours and to evaluate the relationship between this and glutamate levels in the general circulation to allow clinicians to devise focused treatments that prevent metastatic disease.

## Methods

### Cells and reagents

MDA-MB-231 cells (obtained from ATCC) were kept in a humidified incubator at 5% CO_2_, in DMEM with 10% foetal calf serum, 2 mM glutamine and antibiotics. For NMuMG cells, the media was supplemented with 10 μg/ml insulin. For PyMT cells, the media was supplemented with 20 ng/ml EGF and 10 μg/ml insulin. Although MDA-MB-231 cells have previously been reported to express GRM1^31^, there are a number of different strains of this cell line which are known to differ in their invasive characteristics. By using a cDNA library from human brain as a positive control, we have carefully tested the strain of MDA-MB-231 cells that we have used and found that they do not express GRM1 at detectable levels.

ZR75-1 cells (obtained from ATCC) were kept in a humidified incubator at 5% CO_2_, in RPMI medium supplemented with 10% foetal calf serum, 2 mM glutamine and antibiotics. MDA-MB-231, ZR75-1 and in-house lines derived from MMTV-PyMT tumours were routinely tested for mycoplasma contamination using the Beatson Institute’s in-house mycoplasma testing service.

To isolate cell lines from the PyMT model, mice expressing the MMTV-PyMT transgene were culled by CO_2_ euthanasia. Tumours were then surgically excised and minced using a McIlwain tissue chopper. Minced tumour specimens were transferred to DMEM supplemented with 10% foetal calf serum, 20 ng/ml EGF, 10 μg/ml insulin, 2 mM glutamine and antibiotics. PyMT#1 and PyMt#2 cells were generated in this way from two separate mice and maintained in the above medium for a maximum of 20 passages.

To generate GRM3-CRISPR cells, specific guides targeting the exon containing the glutamate binding region were designed using Zhang’s laboratory algorithm (http://crispr.mit.edu/). CRISPR guides were produced using the GeneArt® CRISPR Nuclease Vector Kit (Life Technologies Cat A21174) and transfected using Amaxa nucleofector 2b (Lonza) according to manufacturer’s protocol. Cells were then sorted with an Aria sorter Z6001 (BD Biosciences). CRISPR cleavage kit was used to verify recombination according to manufacturer instructions. Briefly, primers were designed to amplify a 500 bp amplicon containing the CRISPR site. Cas9 activity will generate a smaller fragment around 400 bp.

To generate rescue cell lines, CRISPR-Ctrl and CRISPR-GRM3 cell lines were transfected with human Flag-GRM3 (Addgene, Plasmid #31798) or empty CMV-Flag plasmid. Stable cell lines were also generated by keeping the transfected cells in presence of 1 μg/ml puromycin for 2 weeks.

LY 367385 (selective GRM1 antagonist), CPPG (group III GRM antagonist), LY 341495 (group II GRM antagonist) and LY 354740 (selective group II GRM agonist) were from TOCRIS. Oligomycin, GM6001 and sulphasalazine were from Sigma. siRNAs were from Dharmacon.

### In vivo experiments

For determination of metabolite concentration in tumour-bearing animals, we used FVB/N female mice (>20 generations inbred), carrying a mouse mammary tumour virus (MMTV) promoter-driven polyoma middle T (PyMT) transgene^38^. Animals were culled at 8, 10, 12 and 14 weeks of age. Blood samples were collected via cardiac puncture following euthanasia using CO_2_. Serum was isolated by allowing blood to clot for 5 min at room temperature and then centrifuging at 10,000 × *g* for 15 min. Samples were then stored at −80 °C prior to metabolite extraction in a buffer containing 50% methanol, 30% acetonitrile, 20% water followed by mass spectrometric analysis by LC-MS using HILIC chromatography with full scan mass spectrometry on a Thermo Q-Exactive instrument, measuring metabolites across a mass range of 75–1000 Da.

For tail vein injection, 0.5 × 10^6^ cells in 100 μl PBS were injected into CD1-*nu/nu* recipient mice (Charles River, UK). Mice were culled when they presented signs of metastasis. LY95 (10 mg/kg/day) or PBS control was administered daily by subcutaneous injection as described in^12^.

All experiments were performed following relevant guidelines and regulations, in accordance to the ethical approval from Glasgow University, under the revised Animal (Scientific Procedures) Act 1986 and the EU Directive 2010/63/EU (PPL 70/8645). Animals were housed in individual cages, ventilated in a barrier facility and containing environmental proactive enrichment.

### Measurement of metabolites in cultured cells

Cells were plated at 10^5^ cells per well in six-well plates. 24 h after plating, culture media was removed and replaced with full medium with or without indicated treatments, or medium deprived of glutamine. Cell growth was monitored and culture supernatants were harvested over a 4 day period and frozen at −80 °C. The concentration of glutamate, glucose, lactate and glutamine in the cell-exposed supernatants was then determined using a 2950 Biochemistry analyser (YSI).

### Determination of invasiveness

Fibroblast-derived ECM were generated as described in^10^, but using human immortalised fibroblast as described in^18^. Cells were seeded into fibroblast-derived ECM in six-well plates 4 h prior to imaging by time-lapse phase contrast microscopy (Axiovert S100; Carl Zeiss Micro-Imaging, 10× objective) in an atmosphere of 5% CO2 at 37 °C, as described in ^39^. Protrusion elongation was measured using Image J software. Cells were depleted of glutamine (with or without the addition of glutamate) for 4 h prior to imaging. Because PyMT cells extend protrusions at both ends of the cell, the total protrusion lengths (i.e., the length of all invasive protrusions emanating from the cell) were combined, whereas for MDA-MB-231 cells only the length of the unique front protrusion in the direction of migration was measured.

Three-dimensional culture of PyMT#1 cells was performed as described in ^40^. 5 × 10^3^ cells were plated into Matrigel. Following plating, cells were incubated in DMEM with or without glutamine and the indicated supplements (glutamate, LY40, or LY95) for 4 days, prior to fixation with 2% paraformaldehyde and permeabilisation with 0.5% triton-X100. For 3D culture of normal mammary epithelial cells, NMuMG cells were plated into Matrigel containing 1.6 mg/ml rat tail collagen I. Following culture in full DMEM for 4 days, the medium was changed for DMEM or DMEM supplemented with 10 μM glutamate or LY354740 (LY40; 50 nM) and the cells were cultured for a further 4 days prior to fixation. Antibodies used for immunofluorescence staining were as follow: anti-Collagen IV antibody (Abcam, ab19808); Donkey anti-Rabbit IgG conjugated with Alexa Fluor 488 (ThermoFisher, A-21206). Alexa Fluor 546 Phalloidin (ThermoFisher, A22283) was used to stain F-actin. Images were acquired using an Olympus FV1000 confocal microscope, 60× objective, 1.4 NA and circularity was quantified using the 'Descriptors' plugin in Image J software.

Organotypic invasion assays were performed as previously described^18^. Briefly, human epidermal fibroblasts were seeded in plugs of rat tail collagen 1 which were allowed to contract in full DMEM (DMEM supplemented with 10% FCS and 2 mM glutamine) for 14 days. 4 × 10^5^ cells were then plated on top of these plugs and cultured for 2 days. Plugs were then transferred to a metal grid and cultured with full DMEM with or without LY95 for 1 week followed by fixation in 4% paraformaldehyde before paraffin embedding. 4 μm sections were then cut and stained using Hematoxylin and Eosin.

### Recycling assays

MDA-MB-231 cells were incubated in serum-free DMEM in the absence of glutamine for 1.5 h, transferred to ice, washed twice in cold PBS and surface-labelled at 4 °C with 0.2 mg/ml NHS-SS-biotin (Pierce) in PBS for 30 min. Cells were transferred to serum and glutamine-free DMEM for 30 min at 37 °C to allow internalisation of tracer. Cells were returned to ice, washed twice with ice-cold PBS and biotin was removed from proteins remaining at the cell surface by reduction with MesNa. The internalised fraction was then chased from the cells by returning them to 37 °C in serum-free DMEM in the absence or presence of 15 μM glutamate, with or without LY40, or LY95 as indicated. At the indicated times, cells were returned to ice and biotin removed from recycled proteins by a second reduction with MesNa. Biotinylated MT1-MMP was then determined by capture-ELISA using Maxisorp (Nunc) plates coated with antibodies recognising human MT1-MMP.

### TIRF microscopy

Cells expressing mCherry-MT1-MMP were seeded onto 3.5 cm glass-bottomed plates coated with either fibronectin (for MDA-MB-231) or Matrigel (for NMuMG) 18 h prior to imaging. Media was changed for DMEM lacking serum and glutamine and cells imaged in an atmosphere containing 5% CO_2_ at 37 °C using either a 100X or 60X Plan Apo TIRF objective mounted onto a Nikon Eclipse Ti microscope. To generate time-lapse movies, cells were imaged every 1.5 s. Glutamate or vehicle control was added as a 10× spike 5 min after image collection had commenced. To determine the rate of vesicle recruitment, Image J software (incorporating the TrackMate plugin) was used to calculate the rate at which mCherry-MT1-MMP positive vesicles arrived at the TIRF field.

### Fluorescent gelatin degradation

Acid washed coverslips (19 mm diameter) were coated with 0.005% w/v poly-lysine for 20 min at room temperature, washed with PBS and coated with 80 μl of 1 mg/ml Oregon green 488-conjugated gelatin (Life Technologies). Coverslips were left in the dark for 20 min, washed and crosslinked with 0.5% glutaraldehyde. Coated coverslips were then blocked with 20 mM glycine, sterilised in 70% w/v ethanol and quenched for 1 h in DMEM before addition of MDA-MB-231 cells. Cells were treated with the indicated treatments for 18 h, fixed and stained with DAPI and Alexa Fluor 555 Phalloidin (Life Technologies). Images were collected using an Olympus FV1000 confocal microscope. To quantify degradation, images were processed using the Image J Threshold and Analyse Particles functions. For each field, the total degradation area (black pixels), was divided by the total number of cells.

### SHG imaging

Collagen second harmonic generation (SHG) images from 4% PFA-fixed organotypic plugs were collected using a LaVision Biotec Trim‐scope with a Coherent Chameleon Ti:Sapphire femtosecond pulsed laser. An excitation wavelength of 890 nm was used for generating SHG signal at 445 nm. A z‐stack encompassing a depth of 10 μm was imaged over a region of 150 μm by 150 μm, with at least seventeen pictures per condition in two independent experiments being taken and analysed using Image J. For each depth, the image was automatically thresholded and the percentage of fibrillar collagen coverage was determined by quantifying the percentage of positive SHG pixels. The % of fibrillar collagen coverage was then plotted for each depth and the area under curve was calculated as a readout of SHG signal.

### Quantitative PCR

Primers for human GRM1, 2, 3, 4, 5 and 7 were from ref.^12^. Sequence of GRM6 (Forward: 5′-CCCAGTCAGCTGAAAAGATCTACA-3′; Reverse: 5′-CGCTTTCGCTTCTGCACATT-3′) and GRM8 primers (Forward: 5′-TGAAACCAACACTTCCTCTACCA-3′; Reverse: 5′-TCCAGGAGTGAATTTTTGCGG-3′) were designed using NCBI PrimerTool.

Primers for mouse GRMs were obtained from the Primer Bank, primer ID are as follows: GRM1 (34328063a1), GRM2 (29611608a1), GRM3 (32469489a1), GRM4 (29611479a1), GRM5 (26346991a1), GRM6 (197333818c1), GRM7 (26343463a1) and GRM8 (116812903c1). All primers, except for GRM8 had validation data available on the website (https://pga.mgh.harvard.edu/primerbank/).

QuantiTect primer assays (Qiagen) were used for mouse Rab27A (Cat no. QT00119392), human Rab27A (Cat no. QT00040054), human GAPDH (Cat no. QT01192646) and mouse β-actin (Cat no. QT01136772) detection. RT^2^ qPCR Primer Assays (Qiagen) were used for mouse xCT (Cat no. PPM 41938 C) and MT1-MMP (Cat no. PPM03611D) detection.

### Data availability

The data supporting the findings of this study are available within the article and its supplementary information files and from the corresponding authors upon reasonable request.

## Electronic supplementary material

Supplementary Information

Peer Review File
