# Supplementary material for: Glutaminolysis drives membrane trafficking to promote invasiveness of breast cancer cells
Source: Nat Commun. 2017 Dec 21;8:2255. doi: 10.1038/s41467-017-02101-2 (PMC5740148; doi:10.1038/s41467-017-02101-2)
Supplement: Supplementary file 1 — Supplementary Information [file 41467_2017_2101_MOESM1_ESM.pdf]

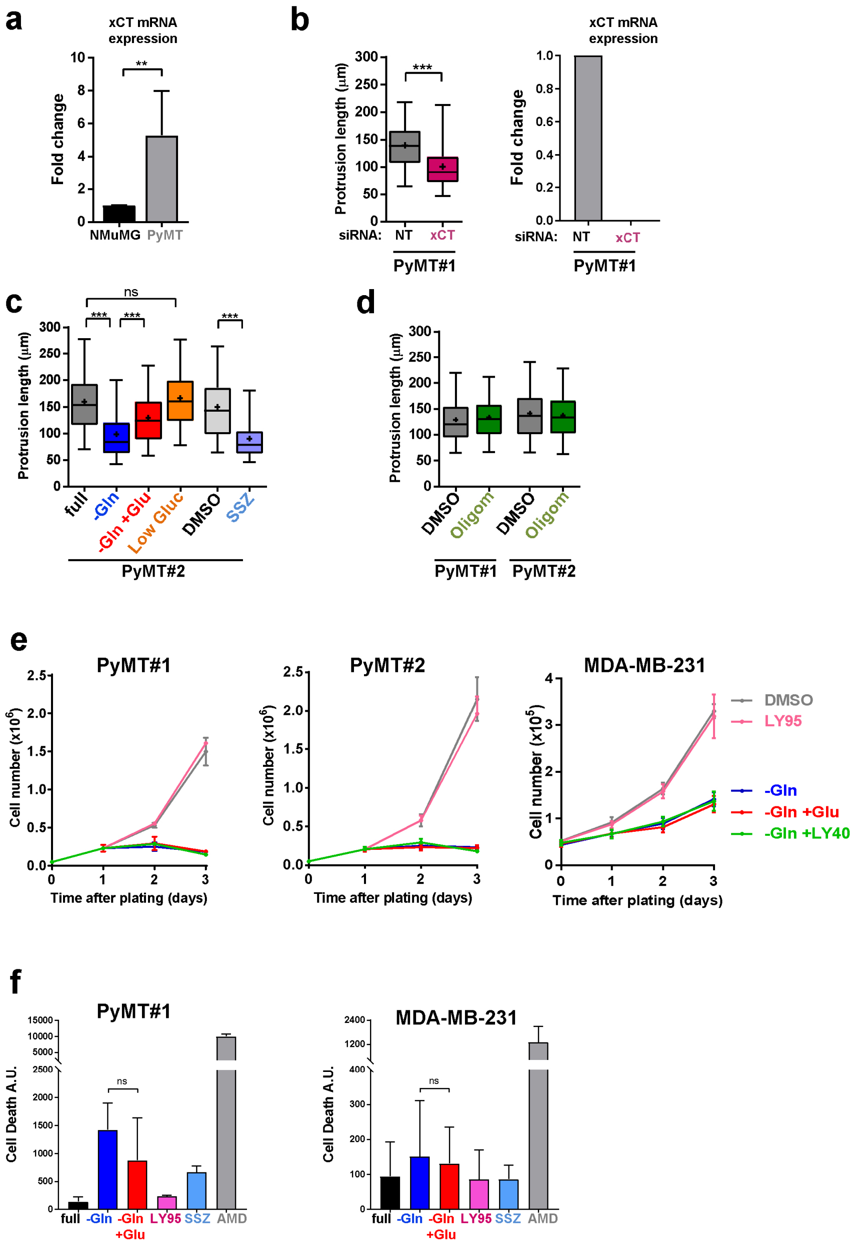

Supplementary Figure 1. Extracellular glutamate drives invasiveness, but does not support cell growth

(a) Comparison of Xc- antiporter expression between mouse normal mammary epithelial cells (NMuMG) and cells from murine MMTV-PyMT driven breast tumours (PyMT). Values are mean ± SEM, n=9 biological replicates. **$p<0.01$ Student's t-test two tailed.

(b) siRNA of Xc- antiporter opposes extension of invasive protrusions in an MMTV-PyMT tumour-derived cell line (PyMT#1). Data are represented as box and whiskers plots (whiskers: 5 - 95 percentile, + represents the mean), 250 cells per condition, n=3 independent experiments *** $p<0.001$ 1way ANOVA, Dunn's multiple comparison test. Confirmation of siRNA suppression of Xc- antiporter is displayed in the right panel.

(c, d) Addition of extracellular glutamate promotes extension of invasive protrusions in PyMT#2 cells. Addition of sulphosalazine (SSZ) opposes invasiveness, whereas oligomycin does not. Data are represented as box and whiskers plots (whiskers: 5 - 95 percentile, + represents the mean), >300 cells per condition, n=3 independent experiments *** $p<0.001$ 1way ANOVA, Dunn's multiple comparison test.

(e, f) Withdrawal of glutamine (-Gln) reduces cell growth and promotes cell death, and this is not reversed by addition of extracellular glutamate (+Glu) or a group II GRM agonist (LY40). Values are mean ± SEM.

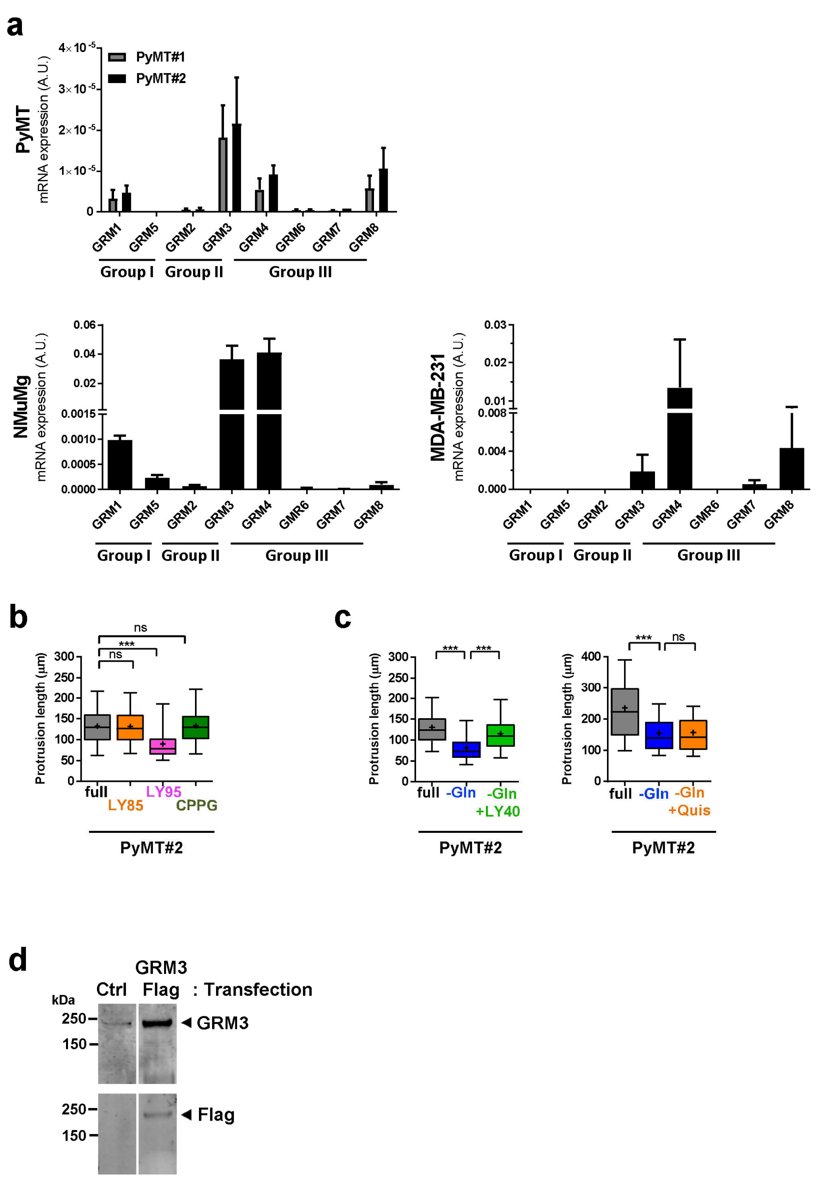

Supplementary Figure 2. Expression of metabotropic glutamate receptors in invasive breast cancer and normal mammary epithelial cells

(a) Expression of members of the GRM family of metabotropic glutamate receptors in PyMT#1, PyMT#2, and MDA-MB-231 cells and normal mammary epithelial cells (NMuMG) was determined using qPCR. The categorisation of the individual GRM receptors into GRM groups I, II & III is denoted on the graphs' abscissae. Values are mean ± SEM. n=3 independent experiments for each cell type

(b) Blockade of group II metabotropic receptors (using LY95) opposes extension of invasive protrusions in PyMT#2 cells, whereas blockade of group I (using LY85) or group III (using CPPG) is ineffective in this regard. Data are represented as box and whiskers plots (whiskers: 5 - 95 percentile, + represents the mean, at least 350 cells per condition), n=3 independent experiments *** p<0.001 1way ANOVA, Dunn's multiple comparison test.

(c) Activation of group II metabotropic receptors (using LY40) in PyMT#2 cells drives extension of invasive protrusions whereas activation of group I receptors (using quisqualate (Quis)) is ineffective in this regard. Data are represented as box and whiskers plots (whiskers: 5 - 95 percentile, + represents the mean), >350 cells per condition, n=3 independent experiments *** p<0.001 1way ANOVA, Dunn's multiple comparison test.

(d) MDA-MB-231 cells were transfected with a vector encoding flag-tagged GRM3 or a control vector. Cells were then surface labelled using NHS-Biotin and surface proteins isolated using streptavidin beads. GRM3 was then determined in these isolates using Western blotting with antibodies recognising GRM3 (upper panel) and the Flag epitope (lower panel).

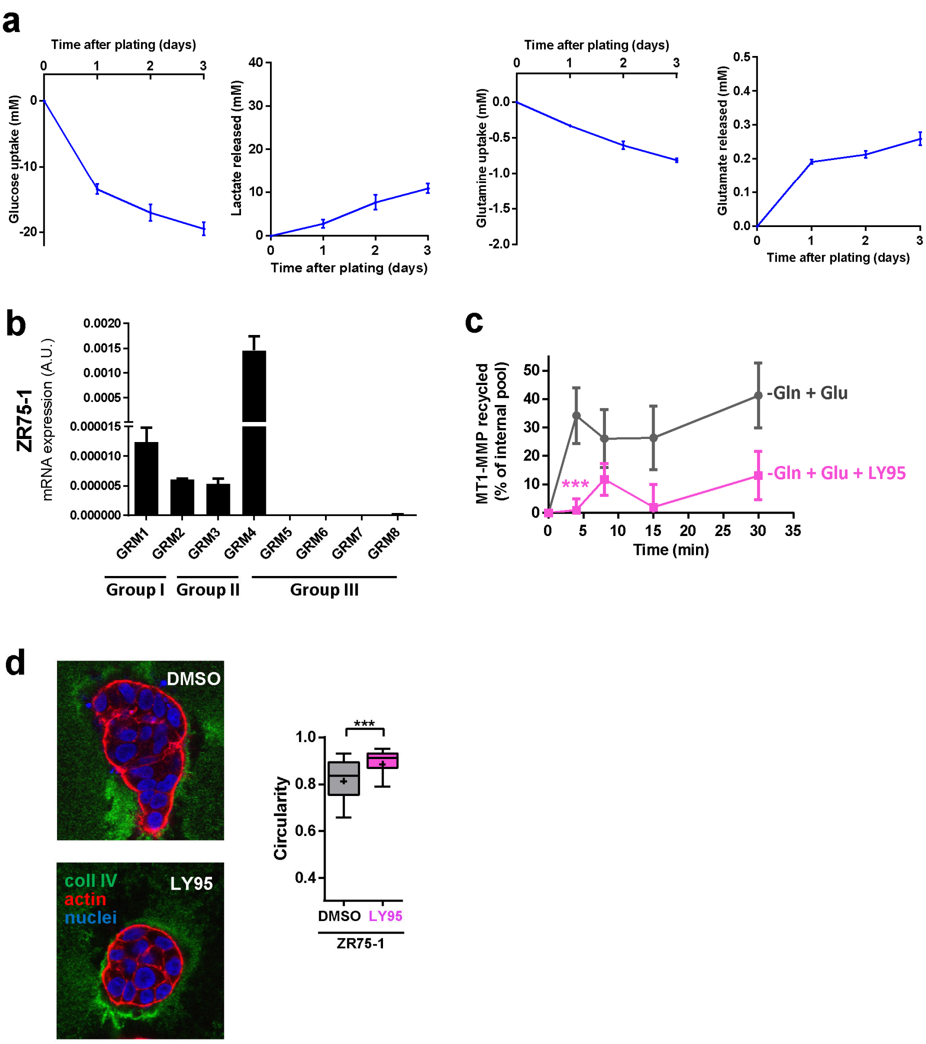

Supplementary Figure 3. Invasive behaviour of ZR75-1 basal type breast cancer cells is dependent on a group II metabotropic receptor

(a) ZR75-1 breast cancer cells consume glutamine and release glutamate. Values are mean ± SEM, n=3 independent experiments.

(b) ZR75-1 cells express group I and group II metabotropic receptors. Values and mean ±SEM, n=2 independent experiments.

(c) Glutamate-driven recycling of MT1-MMP in ZR75-1 cells is opposed by addition of the group II GRM inhibitor, LY95. Values are mean ± SEM. *** $p<0.001$ 2way ANOVA, Dunn's multiple comparison test.

(d) When plated into 3D Matrigel cultures, ZR75-1 organoids assume an invasive morphology and this is opposed by inhibition of group II metabotropic receptors using LY95. Circularity measurements are represented as box and whiskers plots (whiskers: 5 - 95 percentile, + represents the mean), n=3 independent experiments *** $p<0.001$ 1way ANOVA, Dunn's multiple comparison test.

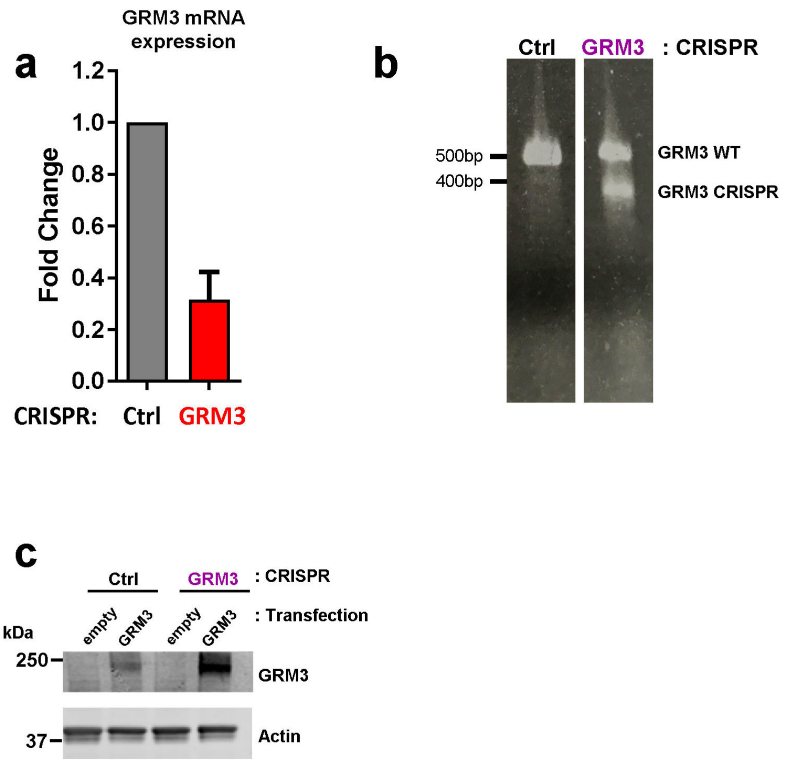

Supplementary Figure 4. CRISPR knockdown and recue of GRM3

(a, b) PyMT#1 cell line in which GRM3 expression had been disrupted by CRISPR gene editing (CRISPR-GRM3) and a corresponding pool that had been transfected with non-targeting CRISPR guides (CRISPR-ctrl) were generated and GRM3 expression in CRISPR-ctrl and CRISPR-GRM3 cells was determined using qPCR. Values are mean ± SEM. N=3 independent experiments. The gel in (b) confirms targeted disruption of the GRM3 gene using the GeneArt Genomic Cleavage Detection Kit (Life Technologies, Cat Number A24372).

(c) CRISPR-Ctrl and CRISPR-GRM3 cells were transfected with a vector encoding human GRM3 or an empty vector control. Cells were lysed and GRM3 expression detected by Western blotting using an antibody recognising human GRM3 (abcam 166608). Actin is used as a loading control.

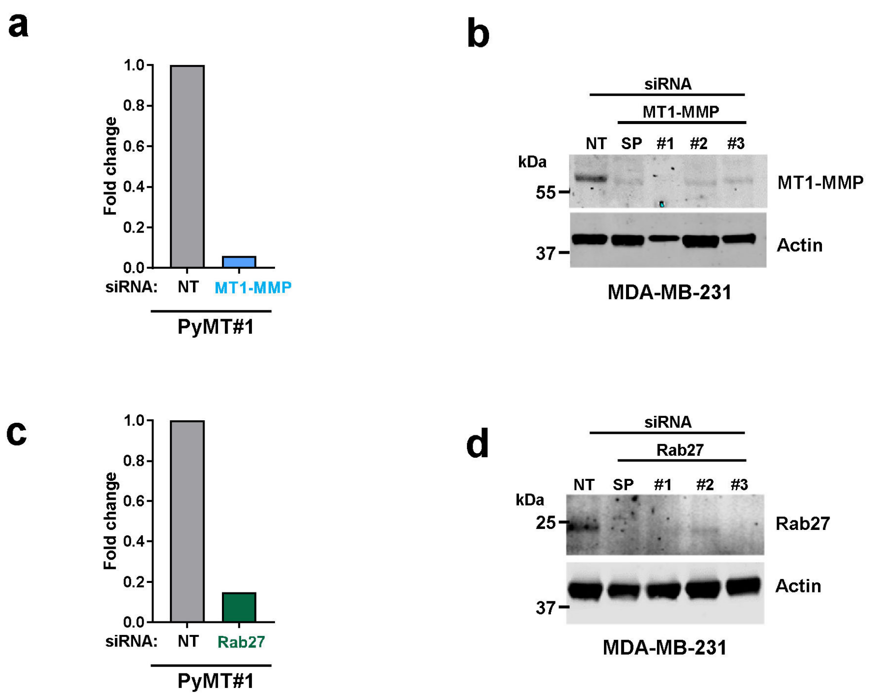

Supplementary Figure 5. Confirmation of siRNA of MT1-MMP and Rab27

PyMT (a and c) and MDA-MB-231 (b and d) cells were transfected with siRNAs targeting MT1-MMP or Rab27 and expression of MT1-MMP and Rab27 detected using qPCR or Western blotting as indicated.

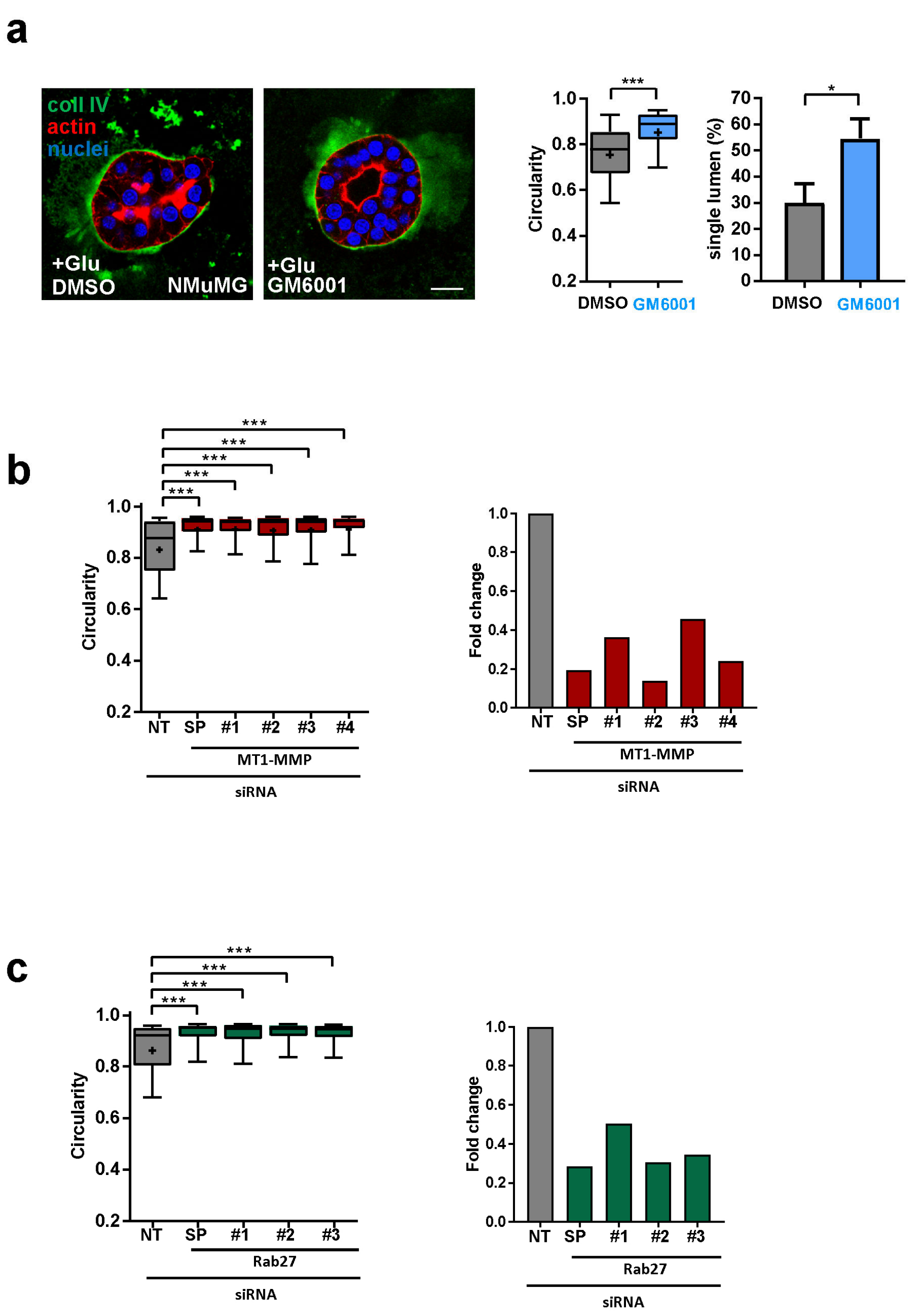

Supplementary Figure 6. Role of MT1-MMP and Rab27 in glutamate-driven invasiveness of normal mammary epithelial cells

(a) Addition of GM6001 (5μM) opposes glutamate-driven basement membrane disruption and lumen filling in normal mammary epithelial cells (NMuMG) in 3D Matrigel cultures. Circularity data are represented as box and whiskers plots (whiskers: 5 - 95 percentile, + represents the mean), n=3 independent experiments *** $p<0.001$ 1way ANOVA, Dunn's multiple comparison test., n=3 independent experiments. The proportion of organoids displaying single lumen was determined by examination of the F-actin distribution across a series of Z-planes using confocal microscopy. Values are mean ± SEM, n=3 independent experiments. *$p<0.05$ 1 way ANOVA Tukey's comparison test.

(b, c) siRNA of MT1-MMP (b) or Rab27 (c) using SMARTPools (SP) or individual siRNA oligos (#1 - #4) opposes glutamate-driven loss of acinar circularity in NMuMG cells. Circularity of NMuMG cell plated into 3D Matrigel cultures was determined as for (a). The efficacy of the siRNA against MT1-MMP (b) and Rab27 (c) was determined using qPCR (right graphs)

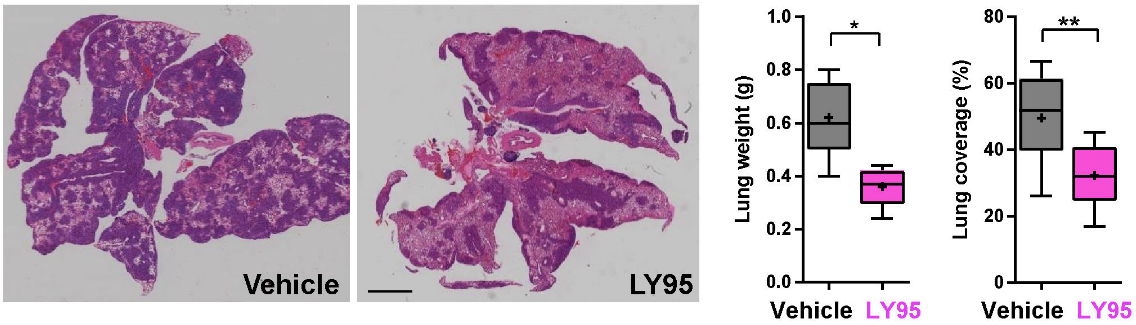

Supplementary Figure 7 Daily administration of a group II metabotropic receptor antagonist opposes lung colonisation

PyMT#1 cells were injected via the tail vein into 6 weeks old CD1 nude female recipient mice (n=5 animals for each group). LY341495 (LY95; 10mg/kg) or PBS control was administered daily by subcutaneous injection. Lung colonisation was assessed by visual inspection, by determination of lung weight and by quantitative assessment of the proportion of tumour-bearing lung tissue across 2 different cross-sections 20µm apart, stained by Hematoxylin and Eosin. Bar, 2 mm. Data are presented as box and whiskers plots (whiskers: 5 - 95 percentile, + represents the mean), representative picture of whole lung stained by Haematoxylin and Eosin is shown for both conditions ** $p<0.001$ *$p<0.05$ 2-sided Mann-Whitney test.
