# Supplementary material for: Glutaminolysis drives membrane trafficking to promote invasiveness of breast cancer cells
Source: Nat Commun. 2017 Dec 21;8:2255. doi: 10.1038/s41467-017-02101-2 (PMC5740148; doi:10.1038/s41467-017-02101-2)
Supplement: Supplementary file 2 — Peer Review File [file 41467_2017_2101_MOESM2_ESM.pdf]

reviewer #1:
(Remarks to the Author):

In manuscript NCOMMS-16-24052-T by Dornier et al, Glutaminolysis drives receptor recycling to promote cancer invasion, the authors examine the role of glutamate metabolism, in particular glyutaminolysis and extracellular glutamate release, in acquiring an aggressive phenotype in breast cancer. They use a one human breast cancer cell line (MDA-MB-231) and a mouse model of breast cancer progression (MMTV-PyMT) to conclude that glutamate release through the system Xc antiporter drives invasive behavior in these cell lines.

The results described are novel and interesting, given that most work regarding glutamatergic signaling in breast cancer has thus far focused on GRM1, and most studies of glutamatergic signaling in other cancers have focused on GRM1, GRM4, or GRM5. Consequently, a role for GRM3 in regulating breast cancer invasiveness would be a novel finding.

Overall, the results are intriguing, but I still have some methodological concerns/questions:

1. Only one human cell line was used (MDA-MB-231). This might not have been as large a concern to me, except that this cell line strikes me as a bit of a mismatch for the MMTV-PyMT model, which more closely models luminal B-type breast cancer. After all, MDA-MB-231 most resembles basal (clinical triple negative). It is not fully clear to me why MDA-MB-231 was chosen as the sole human breast cancer cell line to study with the mouse mammary tumor model instead of a human cell line representing luminal B. It seems to me that at least one other human cell line, preferably one more closely resembling PyMT, should be examined.

2. In Figure S2, no expression of GRM1 is found by qRT-PCR. This is not consistent with previously published data: e.g., Breast Cancer Res Treat (2012) 132:565–573 569 and other publications. No comment on this discrepancy was made.

3. While Figure S1 does clearly show that for the PyMT clones extracellular glutamate doesn't support cell growth, that isn't quite true for MDA-MB-231 cells, which do grow, albeit slower.

4. Was the FBS used in these experiments glutamine- and glutamate-free? If not, why not? Extracellular glutamate and glutamine in FBS can interfere with measurements of glutamatergic signaling, particularly when a competitive antagonist of group I receptors like LY367385 is used.

5. Knockdown of GRM3 is not confirmed by protein anywhere.

6. No protein measurements for any of the GRM receptors was presented, just message levels by qPCR.

7. Statistical analysis appears to be adequate.

Reviewer #2:
(Remarks to the Author):

In the present manuscript Emmanuel Dornier and co-authors demonstrate that cells derived from PYMT-induced mammary gland tumours as well as breast cancer cell line MDA-MB-231 have higher ratio of released glutamate/consumed glutamine than normal mammary epithelial cells. Depriving tumor cells of glutamine or using the inhibitor of Xc antiporter responsible for glutamate export decreased tumour cell ability to form protrusions as a measure of invasiveness. Addition of glutamate restored the ability of cells to form protrusions upon glutamine deprivation.
The authors next demonstrated that inhibiting class II metabotropic glutamate receptors (GRMII) decreased the ability of tumor cells to form protrusions while GRMII agonist rescued the ability to

form protrusions upon glutamine deprivation. This suggested that glutamate promotes invasiveness through GRMII receptors. Disrupting the expression of GRM3 receptor, a GRMII member expressed in studied cells, by CRISPR gene editing inhibited the ability to form protrusions. Treatment of mice injected with PYMT cells intravenously with GRMII inhibitor decreased lung colonisation with these cells.
Finally the authors addressed if glutamate-induced invasiveness is mediated by Rab 27 GTPase-controlled trafficking of MT1-MMP metalloprotease.
Altogether the present results demonstrating the relationship between glutamate, specific type of metabopotropic receptors, GRM3, and invasiveness of breast cancer cells are interesting and quite novel. However the following questions should be addressed:

1. The authors suggest that Xc antiporter is responsible for glutamate release in studied tumour cells. The expression of Xc antiporter in transformed and normal cells should be shown – currently it's "data not shown". It is especially important in light of significantly lower glutamate release by normal cells in comparison with tumour cells.
2. Glutamate rescue of protrusion formation has been only demonstrated for glutamine deprivation. Should be demonstrated for SSZ treatment as well.
3. The authors demonstrate that disrupting the expression of GRM3 by CRISPR gene editing in one of the MMTV-PYMT tumour derived cell lines decreases protrusion formation in vitro. To demonstrate the requirement of GRM3 for the invasiveness in vivo, however, the authors use the treatment with LY95 inhibitor. Comparing the invasiveness of PYMT-WT and PYMT-GRM3 KO cell, which already have been used for in vitro experiments, would be much more specific way of addressing the role of GRM3 in vivo.
4. While in all of the experiments in Figure 1-3, both PYMT and MDA-MB-231 cells are being used, in the experiments in Figure 4a only MDA-MB-231 cells are being used. Is there any particular reason why?
5. The effect of Rab27 knock-down on the formation of protrusions by tumour cells should be demonstrated.
6. To demonstrate that Rab27 glutamate promotes the recycling of MT1-MMP through Rab27, the experiment in Figure 4c should be done in the absence and presence of Rab27 siRNA. Of note, here the control condition of complete media without LY95 inhibitor is missing.
7. In Figure legends for all of the experiments where either protrusion length or circularity is quantified the authors indicate that the results are mean +/- SEM of at least three independent experiments. The number of acini for each condition is indicated. It's not clear if the number of acini was pooled from all three experiments together. If so different types of statistical analysis should be used (the same refers to glutamate measurements in Figure 1b).

Reviewer #3:
(Remarks to the Author):

This manuscript tests the novel hypothesis that invasive tumor cells take up glutamine from their environment, convert it to glutamate, export the glutamate into the extracellular milieu, and finally the exported glutamate activates metabotropic glutamate receptors to stimulate MT1-MMP recycling to the cell surface where it stimulates cancer cell invasion by degrading basement membranes. This is a fascinating story, where an essential nutrient is actually fueling cell movement, rather than simply proliferation. The authors use a variety of three-dimensional in vitro models to complement their metastatic mouse models. Each 3D model (cell-derived matrix, matrigel, collagen, and organotypic dermis) is used appropriately and together they suggest that this pathway could be an important mediator of tumor cell behavior in vivo. My initial enthusiasm was significantly diminished, however, by the lack of basic controls in many instances (such as confirming the efficacy and specificity of the siRNA-mediate protein knockdowns), the absence of any discussion regarding the fact that most cells cannot survive in tissue culture without glutamine

in the media (and how their experiments avoided this potential confounding variable), and a near absence of data supporting their conclusion that glutamate triggers MT1-MMP recycling and increased proteolysis via rab27. Thus, the current manuscript appears very preliminary and more of a starting point for the investigation of this provocative mechanism driving tumor cell invasion. Thus, it seems premature to consider publication at this stage.

Comments:

It is my understanding that L-glutamine is an essential nutrient for most tissue culture cells. Upon removal of L-glutamine, many cell lines/types will cease proliferating and become apoptotic (see: Eagle et al. J. Biol. Chem.1956, 218:607-616 and Eagle H. Science. 1955, 122:501-504 as examples of the extensive investigations into this phenomenon). If this is true, it raises significant doubts about the conclusions the authors made regarding how L-glutamine withdrawal leads to reduced cell invasion. I think this point needs to be explicitly raised in the manuscript and convincingly refuted if the authors are going to conclude that L-glutamine suppresses invasion by preventing the activation of metabotropic glutamate receptors rather than triggering cell death. One way this could be accomplished would be for each experiment which involved complete L-glutamine withdrawal, the number of cells undergoing apoptosis compared to control cells should be reported. This would help mitigate the concern that L-glutamine withdrawal is simply leading to cell death.

The methods section of this manuscript often lacks sufficient information to interpret the presented experimental results. For example, it is not clear what the difference is between the "full DMEM" and "complete media" described on page 11. DMEM comes in many formulations, which was used in this paper? The authors should add a section to the materials and methods making it explicitly clear how the cells were cultured before and during each set of experiments in order for the reader to understand when L-glutmaine was withdrawn and for how long it was withdrawn for. Additionally, all the details required to reproduce their recycling assays should be included as part of this manuscript rather than referring the reader to previous publications.

While it is compelling that MMTV-PyMT tumor-derived cells and MDA-MB-231release more glutamate than the normal mouse mammary epithelial cell line NMuMG, the authors could take advantage of this cell line and use it as an additional control in subsequent experiments to demonstrate the specificity of their proposed mechanism. Specifically, it could be included as a control in Figure 1C, 3A, and 4C.

The efficacy and specificity of the siRNA-mediated protein knockdowns and CRISPR cells should be included. For the siRNA (Rab27 and MT1-MMP), quantifying the reduction in protein and establishing the phenotypic effect for at least two independent siRNA sequences would be beneficial. This will mitigate concerns of off target effects. The loss of protein in the CRISPR-GRM3 should also be confirmed (in addition to the mRNA reduction already documented).

The CRISPR-GRM3 cells could be included in Figure 3 A-C in order to strengthen the conclusion that GRM3 is required for invasiveness.

The data presented in figure 4 is extremely preliminary and does not strongly the support the conclusion that Rab27-dependent recycling of MT1-MMP is mediating the effect of glutamate and GRM3. Some basic questions that could be addressed to strengthen their conclusions include:
-What is the level of MT1-MMP protease activity in response to the different treatments in figure 4C and D?
-Does Rab27 siRNA affect recycling of MT1-MMP (or even the localization)?
-How specific is the disruption of receptor recycling by Rab27siRNA for MT1-MMP? What about other MMPs, or other membrane proteins?
-Does MT1-MMP overexpression rescue invasion in response to glutamine withdrawal and Rab27 siRNA treatment? This experiment could be a way to rapidly establish the sufficiency of MT1-MMP

recycling to the proposed mechanism.
-Why are there no controls included in the recycling assay to increase confidence that this assay is only measuring recycled protein and not global or internalized proteins as well?

In their immunofluorescence images of cells in matrigel, the matrigel looks quite disrupted. Could this be affecting the morphology of the cell clusters? Is the act of fixation disrupting the matrigel?

A schematic depicting the overall mechanism would be extremely helpful.

In their discussion the authors have referenced data that is not included in the manuscript and based some extensive speculation on this unseen data. I think these references should be removed and the discussion refocused on the data presented in the main and supplemental figures. Alternatively, the authors could include the data in the manuscript if they thought it strengthened their conclusions.

Reply to Reviewer's Comments

Reviewer 1:

1. Only one human cell line was used (MDA-MB-231). This might not have been as large a concern to me, except that this cell line strikes me as a bit of a mismatch for the MMTV-PyMT model, which more closely models luminal B-type breast cancer. After all, MDA-MB-231 most resembles basal (clinical triple negative). It is not fully clear to me why MDA-MB-231 was chosen as the sole human breast cancer cell line to study with the mouse mammary tumor model instead of a human cell line representing luminal B. It seems to me that at least one other human cell line, preferably one more closely resembling PyMT, should be examined.

This has been addressed as follows:

We have used MDA-MB-231 cells in our study because basal-type cell lines, and in particular the highly invasive MDA-MB-231 cells, are glutamine auxotrophs and we considered it important to study breast cancer cells with high glutaminolytic activity in the context of the glutamate-driven pro-invasive program we describe in this paper. The reviewer is correct that it is important to deploy an additional human cell line that conforms more to the characteristics of luminal B-type breast cancer. Thus, to ensure that cells with more luminal B-type characteristics are represented in this study, we have also now included experiments conducted with another human cell line, ZR75-1. Consistent with a previous report from the Kaelin lab we show that the luminal B-type ZR75-1 cells are glutaminolytic and that they secrete glutamate. ZR75-1 cells also express GRM3, and they recycle MT1-MMP in a manner that is opposed by the group II GRM antagonist, LY95. Moreover, their ability to form invasive structures in 3D microenvironments is potently opposed by LY95. These data are presented in Fig. S3.

2. In Figure S2, no expression of GRM1 is found by qRT-PCR. This is not consistent with previously published data: e.g., Breast Cancer Res Treat (2012) 132:565–573 569 and other publications. No comment on this discrepancy was made.

This has been addressed as follows:

By using a cDNA library from human brain as a positive control, we have confirmed that the GRM1 primers we use are specifically amplifying GRM1 and that they are able to detect the mRNA for this protein with high sensitivity (not shown). Furthermore, we have now included experiments using the ZR75-1 cells line which we find to express GRM1 at detectable levels (Fig. S3). To directly address the involvement of GRM1 in the invasive response of breast cancer cells we have performed experiments in which we have added a GRM1 agonist, quisqualate to PyMT cells which do express GRM1. These experiments, presented in Fig. 3c and Fig. S2c indicate that GRM1 is not involved in the invasive response to glutamate. We have added a comment to the methods section indicating that our strain of MDA-MB-231 do not express GRM1.

3. While Figure S1 does clearly show that for the PyMT clones extracellular glutamate doesn't support cell growth, that isn't quite true for MDA-MB-231 cells, which do grow, albeit slower.

This has been addressed as follows:

This reviewer is correct that, although withdrawal of *glutamine* ablates growth of MMTV-PyMT cells, the MDA-MB-231 cells continue to grow slowly without this amino acid. However, we believe that the important point made by these data is that addition of *glutamate* to glutamine-starved cells does not restore growth in either cell type.

4. Was the FBS used in these experiments glutamine- and glutamate-free? If not, why not? Extracellular glutamate and glutamine in FBS can interfere with measurements of glutamatergic signaling, particularly when a competitive antagonist of group I receptors like LY367385 is used.

This has been addressed as follows:

This quantity of glutamate in the FBS-containing medium is very low – at a concentration of approximately 10 µM. Although this is sufficient, in theory, to activate GRM3 - in practice this cannot be the case. Indeed, we find that glutamine withdrawal leads to reduced trafficking and invasion whether this is performed in the presence or absence of 10 % FBS. Moreover, addition of GRM3 agonists promote GRM3-dependent trafficking and invasion both with and without FBS. Finally, a GRM1 agonist does not drive invasive responses despite the presence or absence of FBS.

5. Knockdown of GRM3 is not confirmed by protein anywhere.

6. No protein measurements for any of the GRM receptors was presented, just message levels by qPCR.

This has been addressed as follows:

This is an important issue but, in view of the lack of availability of good antibodies and the widely accepted difficulties encountered in measurement of GPCRs by Western blotting, it is not a trivial one to address. By using a surface biotinylation/streptavidin pull-down approach (to capture cell surface proteins) followed by Western blotting, we have been able to confirm the expression of GRM3 protein at the surface of MDA-MB-231 cells and these data are presented in Fig. S2d. However, despite a publication indicating the existence of commercially available antibodies for detecting mouse GRM3, we have been completely unable to find reagents capable of detecting mouse GRM3. Thus in order to validate our CRISPR approach to GRM3 knockdown in MMTV-PyMT cells we have pursued a rescue approach using the human sequence for GRM3 followed by detection with an anti-human GRM3 antibody. Indeed, we have found that, although human GRM3 does not influence the invasiveness of CRISPR-control MMTV-PyMT cells, expression of human GRM3 ***completely rescues*** the invasiveness of CRISPR-GRM3 cells – and this is demonstrable by measurement of the length of invasive protrusion into fibroblast-derived ECM and by

quantification of invasion into organotypic plugs of 3D collagen. These data are presented in Fig. 4b and Fig. 4e of the revision of this paper.

7. Statistical analysis appears to be adequate.

This has been addressed as follows:

We have clearly outlined the statistical tests that we have used in the legends to the figures. We have used Dunn's multiple comparison test for ANOVA and Mann-Whitney test which do not assume parametric distributions. We have also now used box and whisker plots to represent most of our data, as we consider this to be more appropriate than the scatter plots which displayed mean±SEM.

Reviewer 2:

1. The authors suggest that Xc antiporter is responsible for glutamate release in studied tumour cells. The expression of Xc antiporter in transformed and normal cells should be shown – currently it's "data not shown". It is especially important in light of significantly lower glutamate release by normal cells in comparison with tumour cells.

This has been addressed as follows:

This is indeed an important point, particularly in regard of our proposal that it is the acquisition of Xc- antiporter expression that drives glutamate secretion and, in turn, invasiveness. We have now added these data to the paper and they are presented in Fig. S1a. Indeed, the expression of Xc- is at least four-fold higher in transformed invasive MMTV-PyMT cells than it is in normal mouse mammary epithelial cells. We have also used siRNA to suppress levels of the Xc- antiporter and determined the influence of this on extension of invasive protrusions (see Fig. S1b).

2. Glutamate rescue of protrusion formation has been only demonstrated for glutamine deprivation. Should be demonstrated for SSZ treatment as well.

This has been addressed as follows:

We have performed these experiments and the data are now presented in Fig. 1c. Addition of extracellular glutamate restores protrusion formation following SSZ addition in both MMTV-PyMT and MDA-MB-231 cells.

3. The authors demonstrate that disrupting the expression of GRM3 by CRISPR gene editing in one of the MMTV-PYMT tumour derived cell lines decreases protrusion formation in vitro. To demonstrate the requirement of GRM3 for the invasiveness in vivo, however, the authors use the treatment with LY95 inhibitor. Comparing the invasiveness of PYMT-WT and PYMT-GRM3 KO cell, which already have been used for in vitro experiments, would be much more specific way of addressing the role of GRM3 in vivo.

This has been addressed as follows:

We have investigated the invasiveness of CRISPR-control and CRISPR-GRM3 into organotypic plugs. This shows clearly that CRISPR-GRM3 cells have reduced invasiveness (Fig. 4e). To further validate the CRISPR approach, we have expressed human GRM3 in these MMTV-PyMT cells to determine whether this is capable of restoring invasiveness to GRM3 knockout cells. Indeed, this analysis shows that expression of human GRM3 completely rescues invasiveness to CRISPR-GRM3 cells, whilst not affecting the invasive behaviour of CRISPR-control cells (Fig. 4b, e).

As this reviewer suggested, we have also introduced CRISPR-control and CRISPR-GRM3, and these two lines expressing human GRM3 rescue vectors, into the tail vein of nude mice. However, it is clear from this that the extravasation and lung colonisation capacity of MMTV-PyMT cells is not affected by knockout (or rescue) of GRM3. In view of this, we have now re-interpreted our data showing that pharmacological inhibition of GRM3 opposes lung

colonisation and removed any claims concerning a role for GRM3 in metastasis. Thus, we have moved the data showing the influence of LY95 on lung colonisation to the supplementary section (Fig. S7) and we refer to this only in the discussion. The tone of the argument we are now pursuing is as follows: *'This indicates that, although GRM3 directly contributes to invasiveness of breast tumour cells and their ability to breach basement membranes, the contribution made by GRM3 to extravasation and metastatic seeding of disseminated cancer cells is more likely to be mediated via effects of GRM3 inhibitors on other cell type types.'*

We are confident that this modification of our interpretation is appropriate, however we felt that the inclusion of data further supporting an *in vivo* role for extracellular glutamate would reinforce our findings. To do this we have measured levels of glutamate and a number of other metabolites in the circulation of MMTV-PyMT mice as their tumours progress. This analysis clearly indicates that circulating glutamate levels increase significantly in the plasma of tumour-bearing MMTV-PyMT mice by comparison with non-tumour bearing control animals, and this increase correlates temporally with tumour progression and tumour burden (Fig. 1). These data are the first to show that circulating glutamate levels are causally linked to breast tumour progression and support published correlative data indicating that glutamate levels can be elevated in breast cancer patients.

4. While in all of the experiments in Figure 1-3, both PYMT and MDA-MB-231 cells are being used, in the experiments in Figure 4a only MDA-MB-231 cells are being used. Is there any particular reason why?

This has been addressed as follows:

We have now determined the consequences of pharmacological inhibition and siRNA knockdown of MT1-MMP on the extension of invasive protrusions in both MMTV-PyMT and MDA-MB-231 cells. To further reinforce these findings, we have performed the MT1-MMP knockdowns in MDA-MB-231 cells with four independent siRNA sequences. These data are now included in Fig. 5a and Fig. S5.

5. The effect of Rab27 knock-down on the formation of protrusions by tumour cells should be demonstrated.

This has been addressed as follows:

We have now determined the consequences of siRNA of Rab27 on the extension of protrusions from both MMTV-PyMT and MDA-MB-231 cells. To further reinforce these findings, we have performed the Rab27 knockdowns in MDA-MB-231 cells with four independent siRNA sequences. These data are now presented in Fig. 6a and Fig. S5c,d.

6. To demonstrate that Rab27 glutamate promotes the recycling of MT1-MMP through Rab27, the experiment in Figure 4c should be done in the absence and presence of Rab27 siRNA. Of note, here the control condition of complete media without LY95 inhibitor is missing.

This has been addressed as follows:

We have now determined the consequences of siRNA of Rab27 on glutamate-driven recycling of MT1-MMP and these data are presented in Fig. 6c. We have also performed the control experiment requested by this reviewer showing that LY95 opposes glutamate-driven recycling of MT1-MMP and these data are now included in Fig. 6b. To reinforce these data from biotinylation-based recycling assays, we have also used TIRF microscopy to measure MT1-MMP trafficking in both MDA-MB-231 cells and NMuMG. This approach indicates that addition of extracellular glutamate promotes recruitment of MY1-MMP-containing vesicles to the plasma membrane and that this is dependent on Rab27 (Fig. 6d, e & Fig. 7a, b).

7. In Figure legends for all of the experiments where either protrusion length or circularity is quantified the authors indicate that the results are mean +/- SEM of at least three independent experiments. The number of acini for each condition is indicated. It's not clear if the number of acini was pooled from all three experiments together. If so different types of statistical analysis should be used (the same refers to glutamate measurements in Figure 1b).

This has been addressed as follows:

The measurement from all of the experiments were pooled together and all these values are plotted using box and whisker plots to ensure maximum transparency. We now outline this clearly in the reporting checklist.

Reviewer 3:

It is my understanding that L-glutamine is an essential nutrient for most tissue culture cells. Upon removal of L-glutamine, many cell lines/types will cease proliferating and become apoptotic (see: Eagle et al. J. Biol. Chem.1956, 218:607-616 and Eagle H. Science. 1955, 122:501-504 as examples of the extensive investigations into this phenomenon). If this is true, it raises significant doubts about the conclusions the authors made regarding how L-glutamine withdrawal leads to reduced cell invasion. I think this point needs to be explicitly raised in the manuscript and convincingly refuted if the authors are going to conclude that L-glutamine suppresses invasion by preventing the activation of metabotropic glutamate receptors rather than triggering cell death. One way this could be accomplished would be for each experiment which involved complete L-glutamine withdrawal, the number of cells undergoing apoptosis compared to control cells should be reported. This would help mitigate the concern that L-glutamine withdrawal is simply leading to cell death.

This has been addressed as follows:

We have now determined the influence of glutamine withdrawal in the presence and absence of glutamate on the degree of cell death effected under these conditions. Indeed, MDA-MB-231 and PyMT cells are unable to grow in the absence of glutamine, and PyMT cells display moderately increased apoptosis following withdrawal of *glutamine*. However, addition of glutamate did not influence apoptosis nor did it support growth of glutamine-deprived cells, indicating that any role that extracellular *glutamate* might have in cancer progression is mediated via control of the cell migration/invasion machinery and not by promoting cell growth or suppressing apoptosis. These data are now presented in Fig. S1e, f.

The methods section of this manuscript often lacks sufficient information to interpret the presented experimental results. For example, it is not clear what the difference is between the "full DMEM" and "complete media" described on page 11. DMEM comes in many formulations, which was used in this paper? The authors should add a section to the materials and methods making it explicitly clear how the cells were cultured before and during each set of experiments in order for the reader to understand when L-glutmaine was withdrawn and for how long it was withdrawn for. Additionally, all the details required to reproduce their recycling assays should be included as part of this manuscript rather than referring the reader to previous publications.

This has been addressed as follows:

The reviewer is correct that some of our descriptions are inconsistent. As 'full DMEM' and 'complete media' refer to the same thing, we have changed the references to complete media to 'full DMEM throughout. We have added details to the methods of the 3D culture and approaches to indicate when glutamine was withdrawn and glutamate added. Furthermore, we have added a fuller description of the recycling assay to the methods section.

While it is compelling that MMTV-PyMT tumor-derived cells and MDA-MB-231release more glutamate than the normal mouse mammary epithelial cell line NMuMG, the authors could take advantage of this cell line and use it as an additional control in subsequent experiments to demonstrate the specificity of their proposed mechanism. Specifically, it could be included as a control in Figure 1C, 3A, and 4C.

This has been addressed as follows:

NMuMG cells do not extend invasive protrusions and so are not suitable for the type of assays that are presented in Fig. 1c. Correspondingly, when plated onto organotypic plugs, normal mammary epithelial cells sit on top of these and do not invade. (see reviewer Figure R1; below). Finally, in regard of (the original) figure 4c, we have been unable to use the biotinylation-based recycling assay to measure MT1-MMP recycling in mouse cells because we have been unable to obtain a suitable antibody for capture of mouse MT1-MMP. We have, therefore, used an imaging approach to quantify the recycling of MT1-MMP in NMuMG cells. To do this we have used TIRF microscopy to quantify the arrival of MT1-MMP containing vesicles at the plasma membrane, and these data are now presented in Fig. 6d, e and Fig 7a, b.

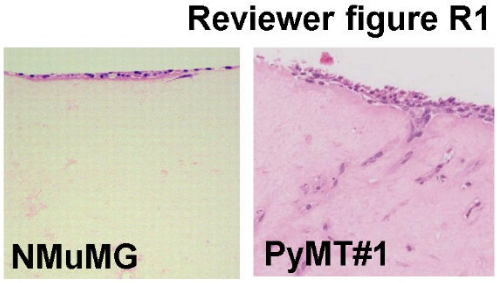

Reviewer figure R1 Normal mammary epithelial cells do not invade into organotypic plugs NMuMG (left panel) and PyMT#1 (right panel) cells were plated onto a collagen plug that had been pre-conditioned by primary human fibroblasts and allowed to invade for 6 days prior to fixation and visualisation of cells using H&E.

The efficacy and specificity of the siRNA-mediated protein knockdowns and CRISPR cells should be included. For the siRNA (Rab27 and MT1-MMP), quantifying the reduction in protein and establishing the phenotypic effect for at least two independent siRNA sequences would be beneficial. This will mitigate concerns of off target effects. The loss of protein in the CRISPR-GRM3 should also be confirmed (in addition to the mRNA reduction already documented).

This has been addressed as follows:

We have performed knockdowns using single oligos targeting both Rab27 and MT1-MMP and confirmed these using Western blots. We have found that single oligo siRNAs that effectively knockdown Rab27 and MT1-MMP also lead to significantly reduced invasive pseudopod length. These data are now included in Fig. 5a, Fig. 6a and Fig S5.

With regard to GRM3, although we have antibodies that recognise human GRM3, we have been unable to find antibodies that recognise mouse GRM3 in either immunofluorescence modalities (flow cytometery or microscopy) or Western blot. However, as discussed in reply

to the other reviewers, we have validated our CRISPR-GRM3 cells using a rescue approach. This analysis indicates that expression of human GRM3 completely rescues invasiveness to CRISPR-GRM3 cells, whilst not affecting the invasive behaviour of CRISPR-control cells (Fig. 4b, e and Fig. S4c).

The CRISPR-GRM3 cells could be included in Figure 3 A-C in order to strengthen the conclusion that GRM3 is required for invasiveness.

This has been addressed as follows:

We have investigated the invasiveness of CRISPR-control and CRISPR-GRM3 into organotypic plugs. This shows clearly that CRISPR-GRM3 cells have reduced invasiveness (Fig. 4e). To further validate the CRISPR approach, we have expressed human GRM3 in these MMTV-PyMT cells to determine whether this is capable of restoring invasiveness to GRM3 knockout cells. Indeed, this analysis shows that expression of human GRM3 completely rescues invasiveness to CRISPR-GRM3 cells, whilst not affecting the invasive behaviour of CRISPR-control cells (Fig. 4e and Fig. S4c).

As this reviewer and reviewer 2 have suggested, we have also introduced CRISPR-control and CRISPR-GRM3, and these two lines expressing human GRM3 rescue vectors, into the tail vein of nude mice. However, it is clear from this that the extravasation and lung colonisation capacity of MMTV-PyMT cells is not affected by knockout (or rescue) of GRM3. In view of this, we have now re-interpreted our data showing that pharmacological inhibition of GRM3 opposes lung colonisation and removed any claims concerning a role for GRM3 in metastasis. Thus, we have moved the data showing the influence of LY95 on lung colonisation to the supplementary section and we refer to this only in the discussion. The tone of the argument we are now pursuing is as follows: *'This indicates that, although GRM3 directly contributes to invasiveness of breast tumour cells and their ability to breach basement membranes, the contribution made by GRM3 to extravasation and metastatic seeding of disseminated cancer cells is more likely to be mediated via effects of GRM3 inhibitors on other cell type types.'*

We are confident that this modification of our interpretation is appropriate, however we felt that the inclusion of data further supporting an *in vivo* role for extracellular glutamate would reinforce our findings. To do this we have measured levels of glutamate and a number of other metabolites in the circulation of MMTV-PyMT mice as their tumours progress. This analysis clearly indicates that circulating glutamate levels increase significantly in the plasma of tumour-bearing MMTV-PyMT mice by comparison with non-tumour bearing control animals, and this increase correlates temporally with tumour progression and tumour burden (Fig. 1). These data are the first to show that circulating glutamate levels are causally linked

to breast tumour progression and support published correlative data indicating that glutamate levels can be elevated in breast cancer patients.

The data presented in figure 4 is extremely preliminary and does not strongly the support the conclusion that Rab27-dependent recycling of MT1-MMP is mediating the effect of glutamate and GRM3. Some basic questions that could be addressed to strengthen their conclusions include:
-What is the level of MT1-MMP protease activity in response to the different treatments in figure 4C and D?

This has been addressed as follows:

We have now performed a substantial body of experimentation to address this issue, and we now feel that the case made for a role for Rab27-dependent recycling of MT1-MMP in GRM3-driven invasion is much stronger. In particular, we have now shown:

a) that addition of glutamate to glutamine-starved MDA-MB-231 cells leads to MT1-MMP-dependent collagen degradation, and this is opposed by LY95. These data are presented in Fig. 5c

b) using second-harmonic generation microscopy we can visualise the quantity of fibrillar collagen in organotypic plugs. We have shown that when MMTV-PyMT cells are plated into organotypic plugs, this leads to a significant degradation of fibrillar collagen within the plug. The ability of these cells to degrade fibrillar collagen is significantly reduced by blockade of group II GRMs with LY95 and by knockout of GRM3 with CRISPR. These data are now presented in Fig. 5d.

c) using TIRF microscopy we have visualised the delivery of MT1-MMP containing vesicles to the plasma membrane of both MDA-MB-231 cells and normal mammary epithelial cells. We are able to quantify movies collected from TIRF microscopy to infer the rate of vesicular delivery of MT1-MMP to the cell surface, and we find that – in both cancer and normal cells – this is driven by addition of glutamate (following glutamine starvation) and that this is opposed by siRNA of Rab27. These data are now presented in Fig. 6d, e and Fig. 7a, b.

-Does Rab27 siRNA affect recycling of MT1-MMP (or even the localization)?

This has been addressed as follows:

We have determined the consequences of Rab27 knockdown on recycling of MT1-MMP in both MDA-MB-231 cells and in normal mammary epithelial cells. This has been performed using biotinylation-based recycling assays in MDA-MB-231 cells, and using TIRF microscopy in MDA-MB-231 cells and normal mammary epithelial cells. In all cases, knockdown of Rab27 reduces glutamate-driven recycling of MT1-MMP. These data are now presented in Fig. 6d, e and Fig. 7a, b.

-How specific is the disruption of receptor recycling by Rab27siRNA for MT1-MMP? What about other MMPs, or other membrane proteins?

This has been addressed as follows:

We have determined the GRM- and Rab27-dependence of recycling of α5β1 integrin in MDA-MB-231 cells. Recycling of α5β1 integrin is promoted by addition of glutamate (although not to the same extent as is recycling of MT1-MMP) and this is opposed by addition of LY95 and by siRNA of Rab27 (Reviewer figure R2).

Reviewer figure R2

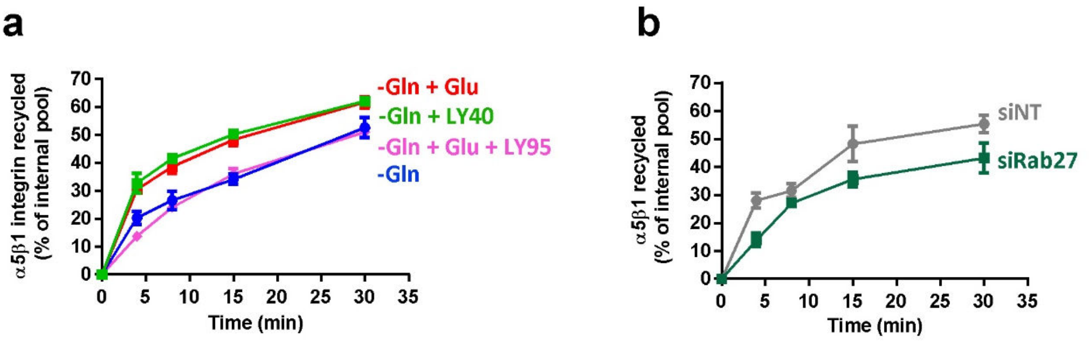

Reviewer figure R2 Addition of extracellular glutamate drives Rab27-dependent recycling of α5β1 in MDA-MB-231 cells. MDA-MB-231 cells were transfected with siRNAs targeting Rab27 (siRab27) or non-targeting control (siNT) (b), or were left untransfected (a). 96 hr following transfection, cells were starved of glutamine for 90 min then surface-labelled with 0.13 mg/ml NHS-S-S-Biotin at 4ºC and internalisation then allowed to proceed for 30 min at 37°C. Biotin remaining at the cell surface was removed by exposure to MesNa at 4°C, and internalised MT1-MMP chased back to the cell surface at 37°C for the indicated times in the absence (-Gln) or presence of 15 µM glutamate (-Gln + Glu), or 50 nM LY354740 (-Gln + LY40) or in full DMEM in the presence of 100 nM LY341495. Cells were then re-exposed to MesNa and biotinylated MT1-MMP determined by capture-ELISA using microtitre wells coated with anti-human α5β1 monoclonal antibodies. The proportion of α5β1 recycled to the plasma membrane is expressed as % of the pool of α5β1 labelled during the internalisation period.

-Does MT1-MMP overexpression rescue invasion in response to glutamine withdrawal and Rab27 siRNA treatment? This experiment could be a way to rapidly establish the sufficiency of MT1-MMP recycling to the proposed mechanism.

This has been addressed as follows:

We have found that levels of MT1-MMP protein in MDA-MB-231 cells are not influenced by activation of GRM3. We conclude from this that GRM3 does not promote invasion by increasing levels of MT1-MMP, but by altering its trafficking/distribution.

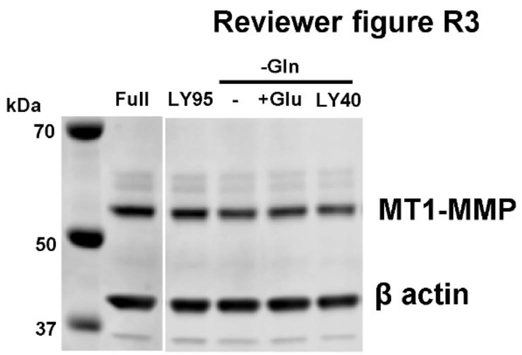

Reviewer figure R3 GRM3 signaling does not influence cellular levels of MT1-MMP protein MDA-MB-231 cells were incubated in full DMEM (full) in the absence or presence of LY95, or starved of glutamine (-Gln) in the absence or presence of glutamate (+Glu) or Ly40 as indicated. Cells were lysed and the cellular levels of MT1-MMP determined using Western blotting with β-actin used a a loading control.

Why are there no controls included in the recycling assay to increase confidence that this assay is only measuring recycled protein and not global or internalized proteins as well?

This has been addressed as follows:

As discussed in the above point, addition of glutamate and/or blockade of LY95 do not influence the global levels of MT1-MMP.

In their immunofluorescence images of cells in matrigel, the matrigel looks quite disrupted. Could this be affecting the morphology of the cell clusters? Is the act of fixation disrupting the matrigel?

This has been addressed as follows:

All the assays to determination the circularity of breast cancer cell spheroids were performed using phase contrast microscopy which does not require fixation. We find that the circularity of invasive and less invasive spheroids is unaffected by fixation.

A schematic depicting the overall mechanism would be extremely helpful.

This has been addressed as follows:

We have now included a schematic as a new figure (Fig. 8).

In their discussion the authors have referenced data that is not included in the manuscript and based some extensive speculation on this unseen data. I think these references should be removed and the discussion refocused on the data presented in the main and supplemental figures. Alternatively, the authors could include the data in the manuscript if they thought it strengthened their conclusions.

This has been addressed as follows:

We have addressed this point by removing references to 'data not shown', and by presenting data discussed in the discussion as figures. For instance, we have now included data describing the alterations to serum glutamate levels that are apparent in MMTV-PyMT tumour-bearing mice. See Fig. 1.

Reviewer #1:
Remarks to the Author:
The authors have adequately addressed my concerns about the first version of the manuscript.

Reviewer #2:
Remarks to the Author:
The authors addressed most of my comments however there are a few outstanding issues:
1. For all of the experiments it would be more appropriate to indicate the times used for treatments in figure legends (including supplementary figures). Currently in most of the cases they are in Materials and Methods and are very difficult to follow.
2. Figure 6b still misses the control with full media.
3. The figure legend for figure 6b states that "full DMEM in the presence of 100 nM LY341495" has been used while it is "-Gln+Glu+LY95" in the figure itself.
4. In the same figure legend – "Values are mean ± SEM., n=3 independent experiments for –Gln and –Gln + Glu; n=2 independent experiments for –Gln + LY40 and LY95" First of all, there is no "-Gln+LY95" condition in the figure. Second, SEM cannot be calculated for n=2.
5. Figure 6c – not clear what media has been used.
6. Page 14, Line 424. "Cells were depleted of glutamine (with or without the addition of glutamine) for 4hr prior to imaging". Should it be "with and without glutamate?"
7. Current title does not reflect the fact that all of the phenotypes are demonstrated in vitro. "Glutaminolysis drives membrane trafficking to promote invasiveness of breast cancer cells", for example, would be more appropriate.
8. Along the same lines the conclusion on page 4, line 131, "However, addition of glutamate did not influence cell death nor did it support growth of glutamine-deprived cells, indicating that any role played by extracellular glutamate in cancer progression is mediated via control of the cell migration/invasion machinery and not by promoting cell growth or suppressing apoptosis" is an overstatement. Although glutamate did not rescue cell survival and proliferation of specific cells used by the authors, the authors cannot conclude that glutamine sensitivity of any other cells cannot be rescued by glutamate. Moreover glutamate ability to rescue various pathways affected by glutamine deprivation may depend on glutamate concentration. In fact it would be helpful if the authors would present the actual metabolites concentrations of metabolites (not A.U.) measured in serum of tumour bearing mice in Figure 1. Are glutamate concentrations comparable to the ones used in the experiments?

Reviewer #3:
Remarks to the Author:
The authors have addressed all of my earlier concerns and I recommend acceptance of their manuscript. I have some very minor new suggestions for the authors that they may wish to consider prior to publication (outlined below). This paper will make an important contribution to our understanding of how protrusions and cell invasion are controlled by the extracellular environment and glutamine metabolism. I look forward to the publication of this ground-breaking work.

Minor suggestions:
-The panels in figure 4C are missing labels as to their treatments (CRISPR, I think).
-The sentence on lines 181 and 182 could be deleted: "However, we have been unable to obtain an antibody capable of detecting mouse GRM3 protein." I think the validation using mRNA can stand on its own without qualification.
-Can the authors indicate in the main text or figure legend the number of experimental replicates used for Fig. 5b? This would increase confidence in their conclusions that all invasion was blocked following GM6001 treatment.

-The second-harmonic images presented in Fig. 5d are not of the same quality as found in the rest of the manuscript. Typically, individual fibers can be detected by SHG (see Wolf et al. Collagen-based cell migration models in vitro and in vivo. Semin. Cell Dev. Biol. 2009. 20(8): 931-941). I recommend the authors obtain higher quality images or simply remove the existing panels from the figure.

Dornier et al., Reply to reviewers' and editor's comments

Replies are in RED

Reviewer#1

The authors have adequately addressed my concerns about the first version of the manuscript.

Reviewer#2

The authors addressed most of my comments however there are a few outstanding issues:

1. For all of the experiments it would be more appropriate to indicate the times used for treatments in figure legends (including supplementary figures). Currently in most of the cases they are in Materials and Methods and are very difficult to follow.

Treatment times have now been included in the figure legends

2. Figure 6b still misses the control with full media.

These data have now been included in a modified figure 6c

3. The figure legend for figure 6b states that "full DMEM in the presence of 100 nM LY341495" has been used while it is "-Gln+Glu+LY95" in the figure itself.

The correct legend is the one on the figure. We have changed the figure legend from "full DMEM in presence…" to "…..glutamine-deprived media supplemented with 15 μM glutamate in the presence of…."

4. In the same figure legend – "Values are mean ± SEM., n=3 independent experiments for –Gln and –Gln + Glu; n=2 independent experiments for –Gln + LY40 and LY95" First of all, there is no "-Gln+LY95" condition in the figure. Second, SEM cannot be calculated for n=2.

We have now included full DMEM and corrected the errors in this figure legend. As advised by this reviewer, we have removed the SEM bars from the -Gln + Glu + LY95 and -Gln + LY40 plots.

5. Figure 6c – not clear what media has been used.

The medium used was –Gln +Glu and this is now clearly indicated on the figure and the legend.

6. Page 14, Line 424. "Cells were depleted of glutamine (with or without the addition of glutamine) for 4hr prior to imaging". Should it be "with and without glutamate?"

Yes. This is a typo and it has now been corrected.

7. Current title does not reflect the fact that all of the phenotypes are demonstrated in vitro. "Glutaminolysis drives membrane trafficking to promote invasiveness of breast cancer cells", for example, would be more appropriate.

The title has been modified as advised by this reviewer.

8. Along the same lines the conclusion on page 4, line 131, “However, addition of glutamate did not influence cell death nor did it support growth of glutamine-deprived cells, indicating that any role played by extracellular glutamate in cancer progression is mediated via control of the cell migration/invasion machinery and not by promoting cell growth or suppressing apoptosis” is an overstatement. Although glutamate did not rescue cell survival and proliferation of specific cells used by the authors, the authors cannot conclude that glutamine sensitivity of any other cells cannot be rescued by glutamate. Moreover glutamate ability to rescue various pathways affected by glutamine deprivation may depend on glutamate concentration. In fact it would be helpful if the authors would present the actual metabolites concentrations of metabolites (not A.U.) measured in serum of tumour bearing mice in Figure 1. Are glutamate concentrations comparable to the ones used in the
experiments?
We have re-phrased this paragraph as follows: “However, addition of glutamate ***at a concentration that modulates invasion*** did not influence cell death nor did it support growth of glutamine-deprived cells, indicating that any role played by extracellular glutamate under ~~cancer progression~~ these ***conditions*** is mediated via control of the cell migration/invasion machinery and not by promoting cell growth or suppressing apoptosis (Supplementary Figure 1e,f).”

Reviewer#3
The authors have addressed all of my earlier concerns and I recommend acceptance of their manuscript. I have some very minor new suggestions for the authors that they may wish to consider prior to publication (outlined below). This paper will make an important contribution to our understanding of how protrusions and cell invasion are controlled by the extracellular environment and glutamine metabolism. I look forward to the publication of this ground-breaking work.

Minor suggestions:
-The panels in figure 4C are missing labels as to their treatments (CRISPR, I think).
These labels have been added to the right panels of figure 4c.

-The sentence on lines 181 and 182 could be deleted: “However, we have been unable to obtain an antibody capable of detecting mouse GRM3 protein.” I think the validation using mRNA can stand on its own without qualification.
We have left this statement. We feel that it offers an explanation for why we can detect the human GRM3 protein, but not the mouse GRM3 protein in the rescue experiments. Furthermore, a number of antibodies are claimed by commercial suppliers to be capable of detecting mouse GRM3. Despite these claims we are unable to reproduce these results.

-Can the authors indicate in the main text or figure legend the number of experimental replicates used for Fig. 5b? This would increase confidence in their conclusions that all invasion was blocked following GM6001 treatment.

These values have been added to the figure legend to figure 5b.

-The second-harmonic images presented in Fig. 5d are not of the same quality as found in the rest of the manuscript. Typically, individual fibers can be detected by SHG (see Wolf et al. Collagen-based cell migration models in vitro and in vivo. Semin. Cell Dev. Biol. 2009. 20(8): 931-941). I recommend the authors obtain higher quality images or simply remove the existing panels from the figure.

As recommended by this reviewer, we have removed these panels from figure 5d
